# Attitudes and experiences of cancer patients toward the provision of audio recordings of their own medical encounter: a cross-sectional online survey

**DOI:** 10.3389/fpsyg.2024.1378854

**Published:** 2024-06-19

**Authors:** Cheyenne Topf, Isabelle Scholl, Pola Hahlweg

**Affiliations:** Department of Medical Psychology, University Medical Center Hamburg-Eppendorf, Hamburg, Germany

**Keywords:** cancer, consultation recordings, patient-centered care, patient information, oncology, cross-sectional online study

## Abstract

**Background:**

The provision of audio recordings of their own medical encounters to patients, termed consultation recordings, has demonstrated promising benefits, particularly in addressing information needs of cancer patients. While this intervention has been explored globally, there is limited research specific to Germany. This study investigates the attitudes and experiences of cancer patients in Germany toward consultation recordings.

**Methods:**

We conducted a nationwide cross-sectional quantitative online survey, informed by semi-structured interviews with cancer patients. The survey assessed participants’ attitudes, experiences and desire for consultation recordings in the future. The data was analyzed using descriptive statistics and subgroup analyses.

**Results:**

A total of 287 adult cancer patients participated. An overwhelming majority (92%) expressed a (very) positive attitude. Overall, participants strongly endorsed the anticipated benefits of the intervention, such as improved recall and enhanced understanding. Some participants expressed concerns that physicians might feel pressured and could become more reserved in their interactions with the use of such recordings. While a small proportion (5%) had prior experience with audio recording medical encounters, the majority (92%) expressed interest in having consultation recordings in the future.

**Discussion:**

We observed positive attitudes of cancer patients in Germany toward consultation recordings, paralleling international research findings. Despite limited experiences, participants acknowledged the potential benefits of the intervention, particularly related to recalling and comprehending information from medical encounters. Our findings suggest that the potential of the intervention is currently underutilized in German cancer care. While acknowledging the possibility of a positive bias in our results, we conclude that this study represents an initial exploration of the intervention’s potential within the German cancer care context, laying the groundwork for its further evaluation.

## 1 Introduction

The provision of audio recordings of their own medical encounters to patients, termed consultation recordings, can help to address information needs of patients by improving understanding, recall, and feeling informed (van der Meulen et al., 2008; Tsulukidze et al., 2014; Barr et al., 2018; Rieger et al., 2018). High information needs are common among cancer patients (Rutten et al., 2005), especially shortly after diagnosis (Matsuyama et al., 2013). Medical consultations are the most important source of information for cancer patients (Rudolph et al., 2015). Ensuring patients receive comprehensible information is crucial, as emphasized in health policy (Bundestag, 2013). A systematic review found that patients with fulfilled information needs experience better health-related quality of life and less anxiety and depression (Husson et al., 2011). However, patients often struggle to accurately recall information provided during medical encounters, with up to 80 percent being forgotten (Ley, 1979; Kessels, 2003; Jansen et al., 2008; van der Meulen et al., 2008; Watson and Mckinstry, 2009; Sherlock and Brownie, 2014). Various reasons have been proposed, including anxiety, pain (Kessels, 2003; Rimmer, 2019) and cognitive deficits (Kessels, 2003; Janelsins et al., 2014; Pendergrass et al., 2018).

One intervention to address this issue is providing patients with consultation recordings. Several international studies have investigated this intervention, predominantly focusing on cancer patients (Tsulukidze et al., 2014), with the majority of research conducted in the United States (Tsulukidze et al., 2014; Elwyn et al., 2017; Grande et al., 2017; Barr et al., 2018, 2021; Joshi et al., 2020; Kwon et al., 2021; Smith et al., 2022), Australia (Lipson-Smith et al., 2016; Moloczij et al., 2017; Hyatt et al., 2018, 2020; Petric et al., 2021; Ryan et al., 2022), and the United Kingdom (Elwyn et al., 2015; Ivermee and Yentis, 2019; Shepherd et al., 2023). Empirical evidence supporting this intervention reveals a wide range of benefits, which help to meet information needs of patients by improving information recall (van der Meulen et al., 2008; Tsulukidze et al., 2014; Barr et al., 2018; Hyatt et al., 2018; Rieger et al., 2018; Dommershuijsen et al., 2019; Kwon et al., 2021; Petric et al., 2021; Shepherd et al., 2023), increasing feelings of being informed (Dommershuijsen et al., 2019; Hack et al., 2021), and enhancing understanding (van Bruinessen et al., 2017; Barr et al., 2018; Hyatt et al., 2018; Kwon et al., 2021; Shepherd et al., 2023). Further benefits include increased patient empowerment (Elwyn et al., 2015; Grande et al., 2017; Hyatt et al., 2020; Smith et al., 2022), facilitation of discussions with family members (Dommershuijsen et al., 2019; Hyatt et al., 2020; Kwon et al., 2021; Petric et al., 2021), heightened satisfaction with care (Pitkethly et al., 2008; Dommershuijsen et al., 2019), improvement in decision-making (Dommershuijsen et al., 2019; Kwon et al., 2021; Smith et al., 2022), and reductions in anxiety and depression (Tsulukidze et al., 2014; Rieger et al., 2018; Smith et al., 2022). Research has explored various ways of implementing this intervention, including patient-led recordings [i.e., patients asking clinicians to record with their own recording device or cell phone (Ryan et al., 2022)], covert recordings (Elwyn et al., 2015; Barr et al., 2018), or provision of recordings, for example via patient-centered smartphone apps (Hyatt et al., 2020; Barr et al., 2021). Nevertheless, the prevalence of consultation recordings for patients remains relatively low, ranging from 15 [United Kingdom (Elwyn et al., 2015)] to 18 [United States (Barr et al., 2018)] percent of patients reporting to have recorded at least one medical consultation in the past. This might be linked to barriers related to the implementation of consultation recordings. Concerns include the potential escalation of patient anxiety from hearing distressing content (Tsulukidze et al., 2014; Moloczij et al., 2017; Hyatt et al., 2018; Hack et al., 2021), a negative impact on the patient-physician relationship (Tsulukidze et al., 2014; Elwyn et al., 2015; Grande et al., 2017; Moloczij et al., 2017; van Bruinessen et al., 2017; Dommershuijsen et al., 2019), healthcare personnel feeling uneasy about being recorded (Tsulukidze et al., 2014; Moloczij et al., 2017; van Bruinessen et al., 2017; Barr et al., 2018; Ivermee and Yentis, 2019), and worries about medico-legal implications (Tsulukidze et al., 2014; Elwyn et al., 2015; Moloczij et al., 2017; van Bruinessen et al., 2017; Barr et al., 2018; Rieger et al., 2018; Ivermee and Yentis, 2019; Joshi et al., 2020; Ryan et al., 2022). Additionally, concerns were raised that consultation recordings could prolong consultations (Tsulukidze et al., 2014; Moloczij et al., 2017). However, consultation recordings have not been found to substantially extend consultation times (Hack et al., 2013; Tsulukidze et al., 2014; Petric et al., 2021). Furthermore, previous studies suggest that patients who received a consultation recording needed less phone calls and had fewer questions later on (Hack et al., 2013; Tsulukidze et al., 2014; Petric et al., 2021). Nevertheless, patients in general express a positive attitude toward consultation recordings (Barr et al., 2018; Dommershuijsen et al., 2019; Ivermee and Yentis, 2019; Hack et al., 2021; Petric et al., 2021; Smith et al., 2022), and express a desire to record consultations in the future (Elwyn et al., 2015; Barr et al., 2018; Dommershuijsen et al., 2019).

Effective implementation of interventions relies heavily on contextual factors, such as culture (Damschroder et al., 2022). However, there is limited research on consultation recordings in Germany. To assess the intervention’s feasibility in the German healthcare context, this study aimed to assess the attitudes and experiences of cancer patients in Germany toward the provision of audio recordings of medical encounters.

## 2 Methods

We report on a cross-sectional nationwide quantitative survey exploring cancer patients’ attitudes and experiences regarding consultation recordings in Germany. Reporting follows the Checklist for Reporting Results of Internet E-Surveys (CHERRIES) (Eysenbach, 2004) (cp. Supplementary file S1).

### 2.1 Participants

Adult cancer patients were eligible to participate. It was planned to include 300 participants in the quantitative survey for pragmatic reasons. As we planned predominantly descriptive data analyses, no *a priori* power analysis was undertaken.

### 2.2 Materials and questionnaires

The development of the survey questionnaire was based on semi-structured qualitative interviews with 11 cancer patients (qualitative methods and results can be found in Supplementary file S2), qualitative interviews with people from the general public in an unpublished preliminary study by the authors, the integrative model of patient-centeredness (Scholl et al., 2014), and additional literature (Elwyn et al., 2015, 2017; Lipson-Smith et al., 2016; Moloczij et al., 2017; Hyatt et al., 2018; Joshi et al., 2020). The survey assessed the experience with, attitudes toward, and desire for consultation recordings. The first sentence of the survey’s first page described to participants what consultation recordings are. When participants indicated that they had experience with consultation recordings, they were asked further questions regarding their experience (e.g., who suggested the recording, how it was done, and with whom they shared their recording). Subsequently, participants were asked about their general attitude toward the intervention on a 6-point Likert scale, ranging from *very negative* (*=1*) to *very positive* (*=6*), followed by 45 questions with statements about the assumed benefits and concerns regarding the intervention, which were assessed on a 6-point Likert scale, ranging from *completely disagree* (*=1*) to *completely agree* (*=6*). When participants indicated that they would like to have audio recordings in the future, they were asked further questions regarding the implementation (e.g., if they would be open to record the consultation with their own phone, if they would listen to the recording, and if they would share it).

Additionally, we employed the German version of the “Affinity for Technology Interaction Short Scale (ATI-S) (Wessel et al., 2019) to evaluate participants’ proclivity for actively engaging with technical systems (Franke et al., 2019). The scale consists of 4 items using a 6-point Likert scale, ranging from *completely disagree* (*=1*) to *completely agree* (*=6*). The ATI-S showed high McDonald’s omega, factor loadings, item difficulty and discrimination, and construct validity (Wessel et al., 2019). Participants’ health literacy was assessed using the German version of the “HLS-EU-Q16” (Pelikan and Ganahl, 2017), consisting of 16 items using a 4-point Likert scale, ranging from *very easy* (*=1*) to *very difficult* (*=4*). To assess participants’ preferred role in treatment decisions, an adapted version of the “Control Preferences Scale (CPS)” (Degner et al., 1997; Rothenbacher et al., 1997; Giersdorf et al., 2003) was used. The instrument measures whether participants prefer the decision-making to be led by either the doctor, the patient, or both, assessed with one item. Additionally, demographic data (e.g., age, gender, language skills, education level) and disease-related information (e.g., cancer diagnosis, level of disease progression) were collected. The questionnaire can be found in Supplementary file S3. The questionnaire was pretested with cancer patients from the study’s advisory board, colleagues from our department, and people from the general population (*n* = 9).

### 2.3 Data collection

We employed a convenience sampling method. Different recruitment strategies were used to disseminate invitations to the survey. Participants were invited via email through the distribution networks of more than a thousand self-help groups for various cancer types, and via social media. In addition, leaflets were distributed at various locations, including conferences and in-and outpatient facilities. Further details on the recruitment process can be found in Supplementary file S4. Before participating in the open online survey, participants were required to provide informed consent electronically and confirm their cancer diagnosis through self-report. The online survey was conducted between June 2022 and April 2023, utilizing the LimeSurvey platform (Limesurvey GmbH, n.d.). Participating patients had the opportunity to receive a 10 Euro incentive.

### 2.4 Data analysis

Only participants who met the inclusion criteria and completed the survey were included in the dataset for analysis. Standardized questionnaires were analyzed according to their manuals. Participants were excluded from analysis if the manuals’ criteria were not met (i.e., certain number of non-responses on questionnaire items). Responses (*n* = 2) to open-ended questions, which were only used in the assessment of experiences, were also included in the analyses.

Data was analyzed using SPSS 27. We primarily calculated descriptive statistics. For all items on attitudes, we calculated frequency distributions, means, standard deviations, and medians from the complete sample. For items on experience with and desire for consultation recordings, we calculated frequency distributions. In addition, we calculated descriptive statistics regarding the attitudes toward consultation recordings for three subsamples: those indicating a desire for consultation recordings, those being undecided, and those having no desire. Furthermore, two hypotheses for subgroup testing were formulated based on results from a previous study (Barr et al., 2018): (1) The attitude toward consultation recordings is more negative with higher patient age, (2) The attitude toward consultation recordings is more negative with lower education level. Regarding hypothesis 1, we compared three age groups: “18 to 39 years,” “40 to 59 years,” and “60 years and older.” Regarding hypothesis 2, we compared three groups with different education levels: “low to intermediate” (i.e., no formal degree or graduation after not more than 11 years at school), “high” (i.e., graduation after more than 11 years at school), and “very high” (i.e., college or university degree). The Jonckheere-Terpstra test, a rank-based nonparametric test, was employed to determine whether there is a statistically significant trend between the ordinal independent variables (age groups, education levels) and the ordinal dependent variable (general attitude) (Sheskin, 2011). Given the presence of two co-primary hypotheses, significance level was set to *p* < 0.025.

## 3 Results of the quantitative survey

### 3.1 Participant characteristics

Two hundred eighty-seven participants met the inclusion criteria and completed the survey. Most participants were female (*n* = 213, 74.2%), between 40 and 59 years old (*n* = 117, 40.8%), and had a very high education level (i.e., college or university degree, *n* = 128, 44.6%). Breast cancer (*n* = 105, 36.6%) and prostate cancer (*n* = 28, 9.8%) were the most frequently reported diagnoses. Additional participant characteristics are presented in Table 1. Additional disease related information of the participants can be found in Table 2.

**Table 1:** Participant characteristics.

**Participant characteristics (*N* = 287)**	***N* (%)**
**Sex**	
Female	213 (74.2)
Male	74 (25.8)
**Age group**	
18–29 years	30 (10.5)
30–39 years	48 (16.7)
40–49 years	53 (18.5)
50–59 years	64 (22.3)
60–69 years	68 (23.7)
70 years and older	24 (8.3)
**Education level** ^**1**^	
Low	16 (5.6)
Intermediate	48 (16.7)
High	92 (32.0)
Very high	128 (44.6)
Other education	3 (1.0)
**Affinity for Technology Interaction (item range 1 to 6)**	
Mean (SD)	3.62 (1.14)
Range	1–6
**German language skills (item range 1 to 10)**	
Mean (SD)	9.72 (0.63)
Range	6–10
**Perceived knowledge of laws regulating audio recordings in Germany**	
Yes	51 (17.8)
No	232 (80.8)
**Level of health literacy**	
Insufficient	84 (29.3)
Problematic	123 (42.9)
Adequate	74 (25.8)
**Preferred level of involvement in treatment decisions**	
I prefer to leave all decisions regarding treatment to my physician.	2 (0.7)
I prefer that my physician makes the final decision about which treatment will be used, but seriously considers my opinion.	26 (9.1)
I prefer that my physician and I share responsibility for deciding which treatment is best for me.	132 (46.0)
I prefer to make the final decision about my treatment after seriously considering my physician’s opinion.	117 (40.8)
I prefer to make the decisions about which treatment I will receive.	10 (3.5)
**Place of residence (German states)**	
Baden-Württemberg	24 (8.4)
Bavaria	37 (12.9)
Berlin	21 (7.3)
Brandenburg	5 (1.7)
Bremen	0 (0.0)
Hamburg	30 (10.5)
Hessen	29 (10.1)
Mecklenburg-Western Pomerania	4 (1.4)
Lower Saxony	34 (11.8)
North Rhine-Westphalia	49 (17.1)
Rhineland-Palatinate	18 (6.3)
Saarland	2 (0.7)
Saxony	16 (5.6)
Saxony-Anhalt	1 (0.3)
Schleswig-Holstein	14 (4.9)
Thuringia	3 (1.0)

Frequencies and percentages not adding up to the total number of participants indicates missing data.

^1^Low, no formal degree or graduation after less than 10 years at school; intermediate, graduation after 10 or 11 years at school; high, graduation after more than 11 years at school; very high, college or university degree.

**Table 2:** Disease related information.

Disease related information (*N* = 287)	*N* (%)
**Cancer diagnosis (multiple answers possible)**	
Breast cancer	105 (36.6)
Prostate cancer	28 (9.8)
Leukemia	24 (8.4)
Ovarian cancer	20 (7.0)
Thyroid cancer	20 (7.0)
Lymphoma	17 (5.9)
Colorectal cancer	14 (4.9)
Skin cancer	7 (2.4)
Lung cancer	6 (2.1)
Cervical cancer	6 (2.1)
Oral cavity and oropharyngeal cancer	3 (1.0)
Kidney cancer	2 (0.7)
Pancreatic cancer	1 (0.3)
Esophageal cancer	1 (0.3)
Other cancer diagnosis^1^	60 (20.9)
**Time since initial diagnosis**	
Less than 1 year ago	46 (16.0)
1 to 5 years ago	123 (42.9)
More than 5 years ago	117 (40.8)
**Current status of disease progression**	
Localized	43 (15.0)
Metastasized	39 (13.6)
In remission/cured	153 (53.3)
Other status^1^	46 (16.0)
**Current state of health (item range 1 to 5)**	
Mean (SD)	2.83 (0.8)
Range	1–5
**Number of medical consultations in the past 6 months**	
Less than 3	99 (34.5)
3–5	84 (29.3)
More than 5	103 (35.9)

Frequencies and percentages not adding up to the total number of participants indicates missing data.

^1^Not further specified.

### 3.2 Attitudes toward the provision of audio recordings of medical encounters

**Figure 1:**
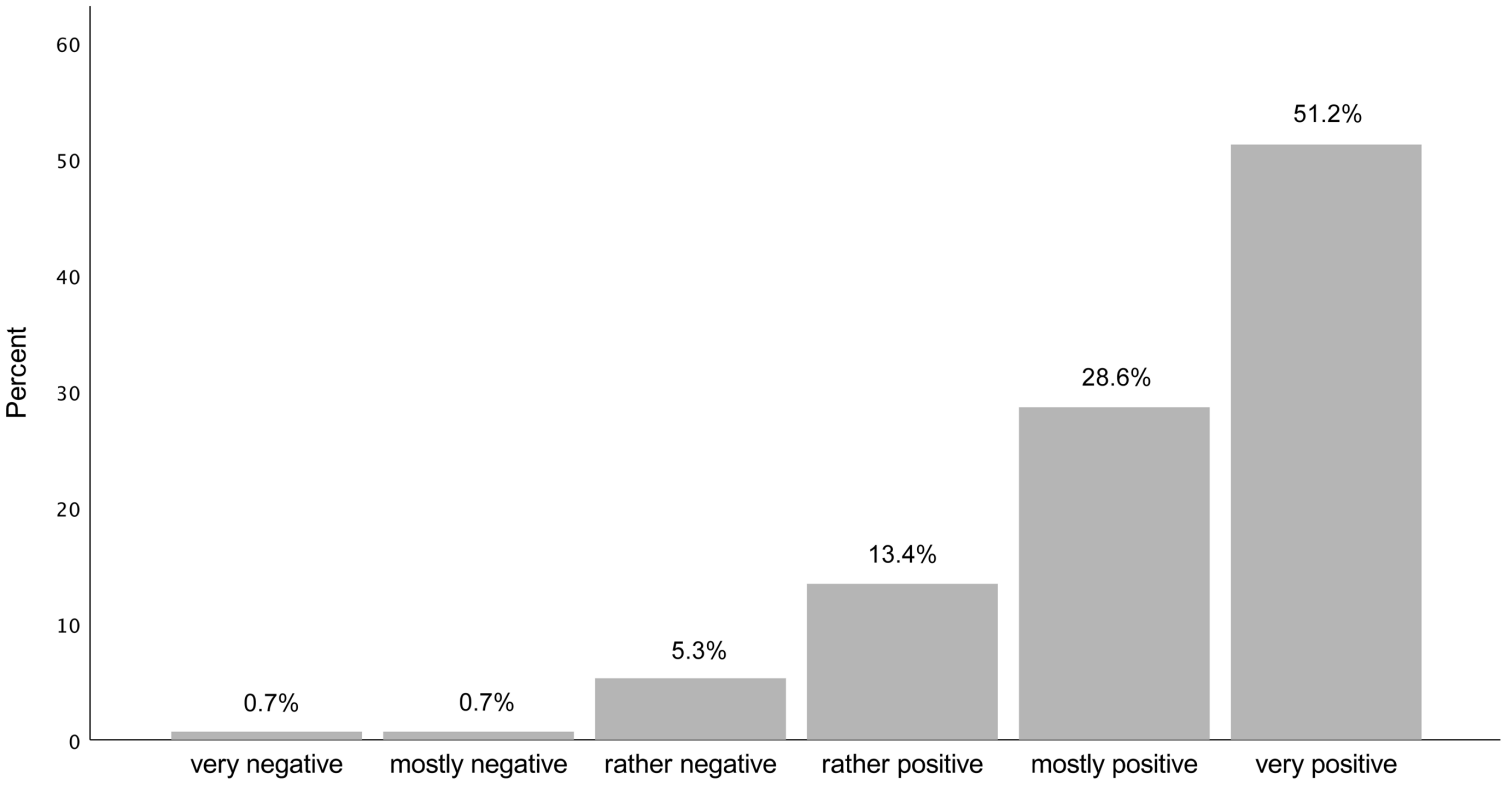
Attitude toward the provision of audio recordings of medical encounters for patients.

The mean of the 6-point Likert scale item on general attitudes toward consultation recordings was 5.22 (SD = 0.99; Median = 6.00), showing very positive attitudes. Most participants (*n* = 265, 91.9%) reported a rather positive, mostly positive or very positive attitude toward consultation recordings (see Figure 1). Table 3 presents statements regarding benefits of consultation recordings and participants’ level of agreement on a scale from *completely disagree* (*=1*) to *completely agree* (*=6*), ranked from highest to lowest mean, Supplementary file 5 includes frequency distribution graphs for all statements about potential benefits. In general, participants demonstrated high to very high agreement to the proposed benefits of consultation recordings (means from 3.87 to 5.74, medians from 4.00 to 6.00). “A consultation recording allows patients to have a better recall of the information discussed” was the item with the highest and “A consultation recording improves the trust between patients and physicians” with the lowest mean. Participants particularly exhibited very high agreement to statements regarding the influence of consultation recordings on recall (X̅=5.74; SD = 0.68; Median = 6.00) and preparation for follow-up appointments (X̅=5.55; SD = 0.86; Median = 6.00). They also concurred that consultation recordings help patients retrospectively verify correct understanding of the information (X̅=5.46; SD = 0.89; Median = 6.00). Additionally, consultation recordings were perceived to enhance the understanding of information (X̅=5.34; SD = 0.91; Median = 6.00). Furthermore, they agreed that consultation recordings are especially helpful in consultations where treatment decisions are made (X̅=5.30; SD = 1.05; Median = 6.00) and in complex and lengthy treatments (X̅=5.29; SD = 1.04; Median = 6.00).

**Table 3:** Levels of agreement toward different statements about benefits of consultation recordings^1^.

A consultation recording…^2^	*N*	Mean (SD)	Median
…allows patients to have a better recall of the information discussed.	284	5.74 (0.68)	6.00
…allows patients to prepare for follow-up appointments (e.g., note down questions).	287	5.55 (0.86)	6.00
…allows patients to retrospectively verify correct understanding of the information.	286	5.46 (0.89)	6.00
…enhances the understanding of information.	287	5.34 (0.91)	6.00
…is especially helpful in consultations in which treatment decisions are made.	286	5.30 (1.05)	6.00
…is especially helpful in complex and lengthy treatments.	285	5.29 (1.04)	6.00
…provides evidence of what was said and done.	285	5.28 (1.01)	6.00
…allows patients to share information with their relatives.	285	5.17 (1.00)	6.00
…is especially helpful when starting or changing a treatment.	284	5.13 (1.17)	6.00
…allows patients to ensure that the physician has understood them correctly.	286	5.08 (1.07)	5.00
…allows a better adherence to medical instructions.	280	5.00 (1.07)	5.00
…is especially helpful for people with language barriers.	270	4.97 (1.15)	5.00
…allows patients to compare their treatment options and make the best decision.	286	4.97 (1.14)	5.00
…should also be conducted when the diagnosis is communicated during the consultation.	285	4.91 (1.47)	6.00
…is especially helpful for people with cognitive deficits.	269	4.84 (1.31)	5.00
…is especially helpful for older people.	279	4.81 (1.18)	5.00
…allows relatives to provide better support to the patient.	281	4.81 (1.15)	5.00
…allows patients to share information with other healthcare professionals.	284	4.76 (1.24)	5.00
…encourages patients to engage with their diagnosis.	282	4.71 (1.17)	5.00
…is helpful for treatment planning.	277	4.71 (1.29)	5.00
…provides protection for patients and physicians.	273	4.58 (1.42)	5.00
…improves the quality of communication.	278	4.55 (1.25)	5.00
…facilitates patients’ active and self-responsible managing of their disease.	285	4.54 (1.28)	5.00
…provides evidence in case of malpractice.	275	4.48 (1.43)	5.00
…facilitates an equal collaboration between patient and physician.	275	4.41 (1.20)	4.00
…allows physicians to be more responsive of concerns and needs of patients.	267	4.38 (1.12)	4.00
…leads to physicians taking their patients more seriously.	271	4.31 (1.22)	4.00
…should be made even in brief consultations with little amount of new information.	287	4.17 (1.48)	4.00
…improves the trust between patients and physicians.	263	3.87 (1.13)	4.00

^1^Answers were assessed on a 6-point Likert scale, ranging from completely disagree (=1) to completely agree (=6); ^2^Items are ordered from highest to lowest agreement.

However, participants somewhat agreed with the proposed concerns about consultation recordings (see Table 4 and Supplementary file S6), although this agreement was not as strong as that related to the proposed benefits. Their primary concerns centered around consultation recordings putting pressure on physicians (X̅=3.77; SD = 1.34; Median = 4.00) and physicians being reserved and less open during the consultations (X̅=3.72; SD = 1.45; Median = 4.00). Lesser concerns included the physician-patient-relationship becoming more formal (X̅=3.47; SD = 1.35; Median = 4.00) and physicians solely referring them to the consultation recording if questions arose after the consultation (X̅=3.45; SD = 1.28; Median = 3.00). Furthermore, participants expressed less concern that consultation recordings would be used as evidence against physicians (X̅=3.44; SD = 1.48; Median = 3.00) and that relatives could pressure patients into allowing them to listen to their consultation recordings (X̅=3.41; SD = 1.43; Median = 3.00).

**Table 4:** Levels of agreement toward different statements about concerns regarding consultation recordings.

I am concerned… ^1^	*N*	Mean (SD)	Median
…that a consultation recording would put pressure on physicians.	281	3.77 (1.34)	4.00
…that physicians would be reserved and less open if consultations were recorded.	283	3.72 (1.45)	4.00
…that the physician-patient-relationship would be more formal if consultations were recorded.	278	3.47 (1.35)	4.00
…that physicians would refer to the recording of the last consultation if any questions came up afterwards.	284	3.45 (1.28)	3.00
…that a consultation recording would be used as evidence against physicians.	264	3.44 (1.48)	3.00
…that relatives could pressure patients into allowing them to listen to their consultation recording.	278	3.41 (1.43)	3.00
…about confidentiality and data protection if consultations were recorded.	283	3.27 (1.57)	3.00
…that listening to the consultation recording would be a psychological burden for patients.	279	3.12 (1.32)	3.00
…about patients perceiving a recording device as stressful during consultations.	282	3.00 (1.35)	3.00
…that patients would be reserved and less open if consultations were recorded.	285	2.99 (1.41)	3.00
…that the technical requirements for making consultation recordings do not exist.	277	2,75 (1.55)	3.00
…that the trust between patients and physicians would decrease if consultations were recorded.	262	2.65 (1.24)	3.00
…that a consultation recording puts too much responsibility on patients.	281	2.60 (1.27)	2.00
…that recording consultations is too complicated for physicians.	282	2.59 (1.44)	2.00
…that recording consultations is too complicated for patients.	283	2.53 (1.36)	2.00
…that the quality of the communication decreases through a consultation recording.	276	2.39 (1.32)	2.00

^1^Answers were assessed on a 6-point Likert scale, ranging from completely disagree (=1) to completely agree (=6); ^2^Items are ordered from highest to lowest agreement.

### 3.3 Association between age and education level with attitudes toward consultation recordings

Descriptively, 18 to 39 year-old participants showed a Median of 6.00 regarding their general attitude toward consultation recordings, 40 to 59 year-olds of 5.50, and people 60 years and older of 5.00. In the Jonckheere-Terpstra test, we found a statistically significant trend toward more negative attitude with rising age in this sample (T_JT_ = 11213.500, *z* = −2.838, *p* = 0.005).

Regarding education levels, the medians for the general attitude item in the three groups were as follows: Median = 5.00 for low to intermediate education level, Median = 6.00 for high education level, and Median = 6.00 for very high education level. The Jonckheere-Terpstra test did show no statistically significant trend in the general attitudes with rising educational level in this sample (T_JT_ = 13275.000, *z* = 1.136, *p* < 0.256).

### 3.4 Experience with consultation recordings

Fifteen participants (5.2%) reported having experience with consultation recordings. Among them, seven (46.7%) had recorded on multiple occasions and four (26.7%) covertly. For the following items, multiple answers could apply. Recordings had been initiated by patients themselves (*n* = 13, 86.7%) or by either a physician, a friend, or a spouse (each *n* = 1, 6.7%). In all instances, consultations had been recorded with a cell phone – either with the patients (*n* = 14, 93.3%) or the accompanying person’s (*n* = 2, 13.3%). Most participants (*n* = 14, 93.3%) had listened to the consultation recording after the encounter – either by themselves (*n* = 8, 57.1%), with their family (*n* = 4, 28.6%), their friends (*n* = 4, 28.6%), their spouse (*n* = 3, 21.4%), their physician (*n* = 1, 7.1%), or another person (*n* = 1, 7.1%).

### 3.5 Desire for consultation recordings in the future

When asked if they wanted consultation recordings in the future, 193 of the 287 participants (67.2%) answered “yes,” 73 (25.4%) answered “maybe,” and 21 (7.3%) answered “no.” Among those who answered “yes” or “maybe,” 220 (82.7%) would be open to recording the encounter with their own cell phone, while 46 (17.3%) would not. Among those who had prior experience with consultation recordings, 13 (86.7%) answered “yes” and two (13.3%) “maybe.” All of these (*n* = 15, 100%) would be open to recording the encounter with their own cell phone.

Two hundred twenty-seven participants (85.3%) expected they would listen to the consultation recordings, 37 (13.9%) maybe, and two (0.8%) would not. Of those expecting to listen, all (*n* = 227, 100.0%) (rather, mostly, or completely) agreed that they would want to listen to what their physician said, 195 (86%) to what they said themselves. Furthermore, 193 participants (72.5%) assumed that they would listen to the recording together with relatives, nine (3.4%) would let their relatives listen to the recording on their own, 40 (15.0%) would not share the recording with relatives, and 24 (9.0%) did not answer this question.

We descriptively analyzed the attitudes regarding consultation recordings separately for those wanting consultation recordings in the future (*n* = 193), those being undecided (*n* = 73), and those not wanting them (*n* = 21) in an explorative approach. On average, those wanting consultation recordings reported more positive general attitudes, higher agreement with potential benefits, and lower agreement with concerns. Those participants who would “maybe” want consultation recordings in the future seemed to build the middle ground, and those declining a wish for consultation recordings reported rather neutral attitudes. However, the third subsample was very small, with only 21 participants indicating no desire for consultation recordings. Detailed results are shown in Supplementary file S7.

## 4 Discussion

In this initial exploration of cancer patients’ views on consultation recordings in Germany, we identified predominantly positive attitudes and high level of agreement regarding potential benefits. Concerns related to consultation recordings were also reported, albeit with less emphasis. Participants viewed encounters involving decisions about complex and burdensome treatment regimens as particularly suitable for recording. Older age was associated with less favorable attitudes toward consultation recordings in our sample. While a minority of participants in our sample had prior experiences with consultation recordings, the majority expressed interest in having consultation recordings in the future. Positive attitudes toward consultation recordings seem to be associated with a desire for future use.

In relation to participants’ positive attitudes toward consultation recordings, our findings align with existing research (Barr et al., 2018; Dommershuijsen et al., 2019; Ivermee and Yentis, 2019; Hack et al., 2021; Petric et al., 2021; Smith et al., 2022). Our study also supports previous research indicating that patients value consultation recordings as a suitable solution to the challenge of recalling and comprehending information from medical encounters (van der Meulen et al., 2008; Tsulukidze et al., 2014; van Bruinessen et al., 2017; Barr et al., 2018; Hyatt et al., 2018; Rieger et al., 2018; Dommershuijsen et al., 2019; Kwon et al., 2021; Petric et al., 2021; Shepherd et al., 2023). Consequently, we can conclude that participants would appreciate consultation recordings as a means to address their information needs. Moreover, we found that consultation recordings were expected to be particularly beneficial in contexts involving treatment decisions, which is consistent with prior research demonstrating that consultation recordings are useful for decision-making (Dommershuijsen et al., 2019; Kwon et al., 2021; Smith et al., 2022). It is noteworthy that in our sample, similar to findings from previous research (Barr et al., 2018), older patients seem to be less in favour of consultation recordings. If age is the cause of the more negative attitudes or a proxy for another cause, it needs to be evaluated in subsequent studies. At the same time, both our study and previous research suggest that consultation recordings could be particularly beneficial for older patients (Dommershuijsen et al., 2019). Nevertheless, some participants expressed concerns about consultation recordings, particularly regarding a potential negative shift in the openness of the physician and in the physician-patient-relationship. Those concerns were also found in previous research (Tsulukidze et al., 2014; Elwyn et al., 2015; Grande et al., 2017; Moloczij et al., 2017; van Bruinessen et al., 2017; Dommershuijsen et al., 2019). Nevertheless, it is worth noting that the overall level of concern about these issues was relatively low, suggesting that participants believe the benefits of consultation recordings outweigh potential disadvantages.

Only a small percentage of participants reported having prior experience with consultation recordings (either overt or covert). This highlights that consultation recordings are not yet widely utilized in Germany, consistent with findings from international studies (Elwyn et al., 2015; Barr et al., 2018). Notably, a considerable amount of those who had shared the recording did so with a relative, emphasizing the potential role of consultation recordings for facilitating discussions and information sharing with the patient’s support network. This observation aligns with findings from previous studies (Dommershuijsen et al., 2019; Hyatt et al., 2020; Kwon et al., 2021; Petric et al., 2021). Furthermore, our study revealed that in almost all cases, participants took the initiative to record the medical encounter with their own cell phone, showcasing an accessible approach to this intervention. The majority of participants also expressed a desire to have consultation recordings in the future, with most of them considering recording the encounter with their own cell phone. This underscores the potential of consultation recordings to not only improve patient outcomes such as recall and comprehension of medical information but also to promote patients’ engagement in their own healthcare, consistent with findings from previous research (Elwyn et al., 2015; Grande et al., 2017; Hyatt et al., 2020; Smith et al., 2022).

The overwhelmingly positive attitudes of our sample, coupled with the expressed desire for consultation recordings in the future, underscores its potential for the German healthcare system. However, the limited experiences of our sample suggests that this potential remains largely underutilized in German cancer care. This could be attributable to physicians in Germany being less open toward technological and digital innovations in general and viewing changes in power structure (e.g., empowering patients) as less favorable than in other countries, thus slowing the adoption of technological innovations in Germany (Safi et al., 2018; Hansen et al., 2019). Furthermore, the link between attitudes and desire to use consultation recordings warrants further exploration.

Despite comprehensive international research on consultation recordings and their well-documented benefits and effects, their applicability to the German healthcare system remains uncertain. Future research, including randomized controlled trials, should delve deeper into the effects within this context and consider diverse perspectives, such as those of relatives, health professionals, and healthcare institutions.

A major strength of our study is that it presents the first investigation of consultation recordings in the German healthcare setting, therewith closing a relevant research gap. Additional strengths include the preceding qualitative interviews that informed the development of our quantitative questionnaire. However, our study is not without limitations. First, selection bias, which limits the generalizability and validity of our results, is anticipated, particularly given that our sample was obtained through self-help groups and patient organizations. This suggests a potential bias toward a more favorable disposition toward patient-centered interventions. Additionally, our sample comprised relatively young and female participants with a high level of education. It is also presumed that due to the online recruitment of participants, individuals with lower technological competence might be underrepresented. This might have contributed to a bias toward positive attitudes about consultation recordings, which might have led to over estimating the feasibility of the intervention. Moreover, the majority of our sample consisted of individuals expressing their attitudes toward an intervention with which they lack personal experience. Second, this study focused on descriptive analyses. We therefore did not conduct power analyses, which limits the external validity of our results. Third, data collection occurred during the peak period of the COVID pandemic, when patients were often alone in medical consultations and were possibly more inclined to welcome an “additional pair of ears,” for which consultation recordings could have been used. Additional limitations include the lack of investigation into reasons for non-participation and the absence of a standardized and psychometrically sound questionnaire to assess attitudes toward consultation recordings.

In conclusion, this study represents an initial exploration of the intervention’s potential within the German cancer care context, laying the groundwork for its further evaluation.

## Data availability statement

The raw data supporting the conclusions of this article will be made available by the authors, without undue reservation.

## Ethics statement

This study involving humans were approved by the Psychological Ethics Committee of the Center for Psychosocial Medicine of the University Medical Center Hamburg-Eppendorf. This study were conducted in accordance with the local legislation and institutional requirements. The participants provided their written informed consent to participate in this study.

## Author contributions

CT: Investigation, Writing – original draft, Formal analysis. IS: Writing – review & editing, Supervision, Project administration, Methodology, Funding acquisition, Conceptualization. PH: Writing – review & editing, Supervision, Project administration, Methodology, Formal analysis, Conceptualization, Funding acquisition.
