# Supplementary material for: Attitudes and experiences of cancer patients toward the provision of audio recordings of their own medical encounter: a cross-sectional online survey
Source: Front Psychol. 2024 Jun 19;15:1378854. doi: 10.3389/fpsyg.2024.1378854 (PMC11220273; doi:10.3389/fpsyg.2024.1378854)
Supplement: Supplementary file 2 [file Data_Sheet_2.PDF]

SUPPLEMENTARY FILE 2

Qualitative Semi-Structured Interviews: Methods, Results, and Additional Documents

1 Methods

1.1 Participants

Adult patients with cancer were eligible to participate. It was planned to include 10 participants in the qualitative interviews.

1.2 Materials and questionnaires

The interviews followed an interview guideline that we developed for this purpose (cp. **3.1 Interview Guide)**. The guideline included questions about participants' experiences with consultation recordings and their attitudes towards it. The anonymous questionnaire, provided to participants before their participation, included demographic questions (e.g. age, gender, education level) and questions regarding their cancer diagnoses (cp. **3.2 Demographic Questionnaire**).

1.3 Data collection

Participants were recruited using convenience sampling. Psycho-oncologists working at the outpatient clinic for psycho-oncology at the Department of Medical Psychology of the University Medical Center Hamburg-Eppendorf invited their patients to participate in the study, either in person or via email. Participants received a consent form and demographic questionnaire prior to participation. The interviews were conducted only after they had provided both written and verbal informed consent. Due to restrictions during the Covid-19 pandemic, interviews were held via telephone. The interviews were conducted between February 2022 and March 2022. Participants could receive an incentive of 25 Euro.

1.4 Data analysis

The telephone interviews were audio-recorded, transcribed by an external contractor, and checked and anonymized by the study team. FS, AS (cp. **Acknowledgements**), and CT analyzed the data using qualitative content analysis (Mayring & Fenzl, 2019) using the software MaxQDA (version 20). One of the PIs of the study (PH) repeatedly

provided feedback during the analysis. The data was analyzed inductively, with the selection criteria being 1) concrete statements regarding participants' experiences with consultation recordings or 2) concrete statements regarding participants' attitudes towards consultation recordings (cp. **3.3 Evaluation Plan**). Descriptive statistics of the demographic data was analyzed using SPSS 27.

2 Results

11 participants were interviewed. Participant characteristics and disease related information of the participants are presented in **Table 1.** The interviews were between 22 and 46 minutes (*mean*=34 minutes, SD=8 minutes).

Table 1

Participant characteristics and disease related information (N=11)

	Mean	SD	Min	Max
**Age in years**	47	14.51	23	68
	N		%	
**Sex**				
Female	8		72.2	
Male	3		27.3	
**Education level**[1]				
Low	1		9.1	
Intermediate	2		18.2	
High	2		18.2	
Very high	6		54.5	
**Cancer diagnosis**				
Breast cancer	3		27.3	
Testicular cancer	2		18.2	
Lung cancer	1		9.1	
Leukemia	1		9.1	
Prostate cancer	1		9.1	
Anal cancer (Squamous cell carcinoma)	1		9.1	
Kidney cancer	1		9.1	
Ovarian and uterine cancer	1		9.1	
**Date of initial diagnosis (…years prior to interview)**				
< 1 year	1		9.1	
1-2 years	3		27.3	
2-3 years	3		27.3	

3-4 years	1	9.1
4-5 years	2	18.2
> 5 years	1	9.1
**Current status of disease progression**		
In Remission/cured	10	90.9
Metastasized	1	9.1
**Date of last medical encounter regarding the cancer diagnosis (…months prior to interview)**		
< 1 month	3	27.3
1-2 months	4	36.4
2-3 months	1	9.1
> 3 months	3	27.3
**Number of medical encounters regarding the cancer diagnosis in the past 6 months**		
< 3	2	18.2
3-5	5	45.5
> 5	4	36.4

Notes: [1] low = no formal degree or graduation after less than 10 years at school, intermediate = graduation after 10 or 11 years at school, high = graduation after more than 11 years at school, very high = college or university degree.

Interview participants were found to have no prior experience with audio recordings. When asked about their attitudes, we found a wide range of aspects that participants expected to be influenced by consultation recordings. The complete coding tree is presented in **Table 2**.

Table 2

Codes and code descriptions

ID	Codes
**E**	**Research Question 1 - Experiences**
**E1**	**No experiences**
**E2**	**Already thought about consultation recordings**
**A**	**Research Question 2 - Attitudes**
**A1**	**Expected changes on the part of the patient**
A1.1	Relief during the consultation
A1.2	Helpful for repetition of consultation content
A1.3	Improved recall
A1.4	Enables to verify correct understanding
A1.5	Increased compliance
A1.6	Basis for in-depth exploration by patient
A1.7	Less need for further exploration by patient
A1.8	Verify whether all questions were asked
A1.9	Helpful for preparation for following consultations
A1.10	Helpful for empowered management of the disease by patient
A1.11	Shared decision-making
A1.11.1	*Promotion of shared-decision making*
A1.11.2	*Uncertainty regarding already made decisions*
A1.11.3	*No impact on shared decision-making*
A1.12	Emotional-therapeutic benefit (including coping with the disease)
A1.13	Facilitated exchange between people with cancer
A1.14	Increased insecurity due to the offer of consultation recording
A1.15	Risk for misunderstandings
A1.16	Listening to recording is emotionally burdensome
A1.17	Excessive occupation with the disease
A1.18	No benefit for empowerment
A1.19	No impact on being well informed

**A2**	**Physician-patient communication**
A2.1	Mutually more precise/appropriate/understandable communication
A2.2	Facilitation of communication on an equal footing
A2.3	More empathetic conversation
A2.4	Reduced openness/authenticity in conversation
A2.5	Being excessively referred to consultation recording
A2.6	No impact on communication
A2.7	Disruption of the conversation flow
**A3**	**Physician-patient relationship**
A3.1	Relationship-building
A3.2	Need for physician openness for consultation recordings
A3.3	Need for voluntariness of recording and listening
A3.4	Burden on the relationship
A3.5	Misinterpretation of relationship by patients
A3.6	No impact on the relationship
**A4**	**Patient safety**
A4.1	Facilitation of comparison between physicians
A4.2	Easier detection of misunderstandings/malpractice
A4.3	Evidence of malpractice
A4.4	No impact on patient safety
**A5**	**Expected changes on the part of the physician**
A5.1	Supplement to the intake interview
A5.2	Protection
A5.3	Helpful for improvement in communication/training purposes
A5.4	Helpful for research
A5.5	Risk of the misuse of consultation recording against the physician
A5.6	Listening is exhausting for the physician
**A6**	**Involvement of family members**
A6.1	Easier involvement of family members
A6.2	Easier support from family members

A6.3	Sharing as relief for patients
A6.4	Sharing as a burden for patients
A6.5	Sharing as a burden for family members
A6.6	Concerns about certain topics
**A7**	**Practical-technical implementation**
A7.1	Technical difficulties during recording
A7.2	Technical difficulties during playback
A7.3	Need for simple technical solutions
A7.4	Need for additional consultation transcription
A7.5	High technical effort and costs
**A8**	**Collaboration between physicians/healthcare professionals**
A8.1	Benefit for other healthcare professionals
A8.2	Concerns about sharing with other healthcare professionals
A8.3	Listening is too time-consuming for other healthcare professionals
**A9**	**Different groups of people**
A9.1	Particularly helpful for certain groups of people
A9.2	Helpful for all kind of people
**A10**	**Different consultation occasions/types**
A10.1	Helpful for initial consultations/diagnosis communication
A10.2	Helpful for therapy planning and pre-treatment consultation
A10.3	Helpful for transition to follow-up/discharge
A10.4	Helpful for complex consultations
A10.5	Too burdensome in diagnosis communication
A10.6	Unsuitable for emergency/shock situations
A10.7	Not necessary for routine/control consultations
A10.8	In psychotherapeutic consultations
**A11**	**General conditions in Germany**
A11.1	Concerns about data protection
A11.2	No/low concerns about data protection
A11.3	High bureaucratic effort

A11.4 Digitization

A12 No need for consultation recordings

3 Additional Documents

3.1 Interview Guide

<u>Preparation checklist for interviewer</u>

- Informed consent given?
- Demographic questionnaire received?
- Administrative form for incentives received?
- Two recording devices prepared?

<u>Interview</u>

Introduction

- The interviewer (CT) calls the participant at the agreed-upon time.
- *"Hello [Mrs./Mr. X], my name is Cheyenne Topf. As you know, I am calling for our scheduled telephone interview. Thank you once again for agreeing to participate. The interview will take approximately 30-60 minutes. I have received your consent form, the incentives form, and the short questionnaire. Do you have any further questions? If not, we can proceed with the interview, and as discussed, I will now start the audio recording."*
- Turn on the audio recording device
- *"First, I would need the anonymous code that you provided on the questionnaire. If you do not have it at hand, please answer the following questions:*

    1. *What is the first letter of your mother's first name?*
    2. *What is the first letter of your father's first name?*
    3. *What is the last digit of your house number?*
    4. *What is the last digit of your telephone or mobile number?"*

- *"As you are aware, these interviews are being conducted to gain insight into your perspective as a patient about receiving an audio recording of your medical encounter."*
- *"I will now ask you some questions one by one, and I kindly ask you to share everything that comes to your mind. For this interview, it is important that you feel comfortable expressing your opinions and experiences openly and honestly.*

There are no right or wrong answers. Feel free to share both critical and positive impressions."

Context:

- *"The opportunity to record medical encounters and provide them to patients is more common in other countries such as the USA, UK, and Australia than in Germany. This is why we are conducting this project in Germany."*

Guiding Questions:

1. *When you hear about this, what is your initial impression of taking an audio recording of your medical encounter with you?*

- *“Next, I would like to discuss your ideas on how to implement such a practice.”*

2. *How could these audio recordings be made and provided?*
   a. *In what situations could such recordings be helpful?*

   b. *Who could benefit from such recordings?*

   c. *Why would you listen to the consultation recordings afterward?*

   d. *When and with whom would you listen to the consultation recordings afterward?*

3. *How would you react to the offer of taking such a recording?*

- If the participant hints at previous experiences:

4. *What experiences have you had with recording an encounter with your physician?*

- *“I would now like you to imagine being in a medical encounter and receiving such an audio recording. You can think about past or future encounters.”*

5. *What could be advantageous or helpful about recording medical encounters?*

- Follow up until no more points are mentioned; if the participant shares experiences, ask about what else they can think of.

6. *What might be challenging or disadvantageous about making such recordings?*

- Follow up until no more points are mentioned; if the participant shares experiences, ask about what else they can think of.

7. *What changes would you expect if you were to take a recording of your encounter with your physician?*
    a. *How could the recording change how well-informed you are and how much you know about your condition and treatment?* (Dimension: Patient information)
    b. *How could the recording change your involvement in treatment decisions?* (Dimension: Patient involvement)
    c. *How could the recording change how actively and independently you manage your condition and treatment?* (Dimension: Empowerment)
    d. *How could the recording change the relationship between you and your physician?* (Dimension: Provider-patient relationship)
    e. *How could the recording change communication with your physician?* (Dimension: Provider-patient communication)
    f. *How could the recording change the involvement of your family members?* (Dimension: Involvement of family members)
    g. *How could the recording change collaboration among different healthcare providers?* (Dimension: Coordination and continuity; integration of medical and non-medical care)
    h. *How could the recording affect your patient safety?* (Dimension: Patient safety)

- *"Now that we have discussed your thoughts on these recordings, I would like to ask a few questions about your personal competency with digital devices."*

8. *How confident do you feel in using digital devices such as smartphones, computers, or tablets?*

- Follow up if not mentioned
    a. *Do you know how to send and receive emails?*
    b. *Do you know how to make an audio recording?*
    c. *Do you know how to connect a USB drive to a computer and use the data on it?*
- *"Finally, I would like to ask you:"*

9. *Can you envision using such consultation recordings in the future? Has anything changed about this since the beginning of the interview?*

Summary:

10. *When you think back to everything we have discussed today; what aspects are particularly important to you?*
11. *Is there anything we haven't discussed that must not be overlooked?*

Conclusion:

- Thank you and goodbye
- Turn off the audio recording device

3.2 Demographic Questionnaire

Dear participant,

With this questionnaire, we would like to gather some anonymous information about you, your illness, and your experiences with medical encounters. Most questions can be answered by checking the appropriate boxes. For other questions, you can enter your response in the open text fields. It should take approximately 5 minutes to complete the questionnaire.

All information provided is confidential, and all project team members are bound by confidentiality agreements. In order to link the information from the questionnaire to the telephone interview, we kindly ask you to provide a code below. We will ask for this code again at the beginning of the interview but will not associate it with your identity. The evaluation will be conducted anonymously at the Institute for Medical Psychology at the University Medical Center Hamburg-Eppendorf. We are very grateful for your participation in this project and thank you warmly for your support.

To create an anonymous code for you, we need the following information:

- First letter of your mother's first name:
- First letter of your father's first name:
- Last digit of your house number:
- Last digit of your telephone or mobile number:

<u>Today's Date:</u>
DD MM YYYY

Personal Information
Please provide some information about yourself:

1. Age: ____________________ (in years)
2. Gender:

   ☐ Female ☐ Male ☐ Non-binary/Other ☐ Prefer not to say
3. Highest (Educational) Degree:

   ☐ No formal education

   ☐ Lower Secondary Education

   ☐ Intermediate Secondary Education

   ☐ High School Diploma/General Certificate of Education

   ☐ Technical/Vocational School Diploma

   ☐ College/University Degree

   ☐ Other: (please specify) _______________________________

Information About Your Illness and Experience with Medical Encounters

4. What type of cancer have you been diagnosed with?

    ____

5. When was your initial diagnosis? Please specify the month and year.

    ____

6. What is the current status of your illness? (e.g., localized, metastasized, in remission/"cured")

    ____

7. When was your last medical encounter regarding your cancer diagnosis? Please specify the month and year.

    ____

8. Approximately how many medical encounters about your cancer diagnosis have you had in the last 6 months?
    ☐ Less than 3
    ☐ 3-5
    ☐ More than 5

Thank you for completing the questionnaire!

3.3 Evaluation plan

Qualitative Content Analysis according to Mayring & Frenzl (2019)

Planned on: 06 September 2022, iteratively revised

Planned by: Funda Sahbudak, Alena Schneider, Cheyenne Topf, Pola Hahlweg

Material Definition: Transcripts of audio recordings of 11 interviews with 11 cancer patients conducted as part of the study "Patient-centered cancer care by providing patients with audio recordings of medical encounters - a feasibility study."

Research Questions (RQ):

1) What experiences do cancer patients report regarding consultation recordings?
2) What justifies the attitude of cancer patients towards consultation recordings?

Analytical Approach (Inductive or Deductive): Inductive

Selection Criteria (Precise Definition):

1) Concrete statements regarding the experiences of a participants (PT) concerning consultation recordings. This includes coding statements when a PT reports having no experiences. "Experiences" here refer to knowledge gained through one's own perception and experience of consultation recordings (see https://dorsch.hogrefe.com/stichwort/erfahrung for reference). This may include descriptions of one's own behavior, thoughts, or feelings and may also involve the behavior of others.
2) Concrete statements explaining the attitude of a participant (PT) toward consultation recordings. "Attitude" here refers to the psychological disposition towards consultation recordings, associated with an judgement or expectation (see https://dorsch.hogrefe.com/stichwort/einstellung#search=cfe219c9c04e1f0f5f9d2c271735c848&offset=0). This can be, for example, positive, neutral, negative, undecided, or skeptical. The reasons provided for these specific attitudes will be coded. Additionally, each code will be assigned a direction to indicate whether the reason tends towards a positive or negative attitude.

Level of Abstraction: Concrete statements related to the selection criteria

Analysis Units:

- Coding unit (minimum): meaningful phrase
- Context unit (maximum): entire text section on a topic

- Evaluation unit: Entire transcript
- Multiple coding for different aspects possible
- Multiple coding within one transcript possible

Additional Note: If no experiences are reported throughout the entire transcript, the entire transcript will be coded with the label "no experiences."

Coding steps: Analysis will be conducted in successive stages for each research question.

A) FS and AS read through all the data material to get an overall impression.

B) Patient Experiences (RQ1)

1. FS and AS code 10% of the patient material (=1 transcript; double coding of the same material)
2. FS and AS discuss and compare their respective codings of the first 10% of the data material and the resulting codes, making necessary revisions.
3. Joint discussion within the project team with CT and PH.
4. FS and AS code an additional 40% of the patient material (double coding of the same material)
5. A consensus discussion takes place again between FS and AS.
6. FS and AS code the remaining 50% of the material (double coding of the same material)
7. Joint discussion within the project team with PH.
8. Another round of feedback and consensus discussion between FS and AS. This includes reviewing the code labels for clarity and making any necessary adjustments.
9. Evaluation validation by CT and further discussion within the team (CT, FS, AS) – for handover
10. Thorough review of codes and coded passages with revisions by CT
11. PH checks the code system and has a joint discussion with CT
12. CT incorporates feedback and finalizes the analysis for RQ 1

C) Patient Attitudes (RQ2)

1. AS and FS code 20% of the patient material (= 2 transcripts, double coding of the same material)
2. FS and AS discuss and compare their respective codings of the first 20% of the data material and the resulting codes, making necessary revisions.

3. Joint discussion within the project team with PH and further adjustment of the code system and codings.
4. FS codes the remaining patient material
5. AS reviews the codings and the code system for comprehensibility and consistency and adds if necessary.
6. Another round of feedback and consensus discussion between FS and AS, as well as adjustments to the code system and codings.
7. Evaluation validation by CT and further discussion within the team (CT, FS, AS) – for handover
8. CT checks the codings and the code system (including memos) for comprehensibility and consistency and conducts comprehensive revisions.
9. PH checks the code system and has a joint discussion with CT
10. CT incorporates feedback and finalizes the analysis for RQ 2

References

1. Mayring, P., & Fenzl, T. (2019). Qualitative Inhaltsanalyse. In *Handbuch Methoden der empirischen Sozialforschung* (pp. 633–648). Springer Fachmedien Wiesbaden. https://doi.org/10.1007/978-3-658-21308-4_42
