# Supplementary material for: Attitudes and experiences of cancer patients toward the provision of audio recordings of their own medical encounter: a cross-sectional online survey
Source: Front Psychol. 2024 Jun 19;15:1378854. doi: 10.3389/fpsyg.2024.1378854 (PMC11220273; doi:10.3389/fpsyg.2024.1378854)
Supplement: Supplementary file 3 [file Data_Sheet_3.PDF]

SUPPLEMENTARY FILE 3

Quantitative online survey

Note: This questionnaire was initially formatted as an online survey and has been translated from German.

The provision of audio recordings of medical encounters for patients

Information and Consent Form for Participation in an Online Survey.

Dear Participants,

We would like to understand your thoughts on providing audio recordings of their own medical encounters to patients with cancer. We are particularly interested in the perspectives of patients with cancer and oncologists. Your participation is crucial to this project.

We kindly ask you to complete the following anonymous questionnaire. It includes questions about your experiences and attitudes, as well as some questions about yourself (e.g., age, gender, health status).

Filling out the questionnaire will take approximately 20 minutes. As a token of appreciation for your participation, you can receive a compensation of €10 via bank transfer or a voucher (more information: www.wunschgutschein.de). The personal information required for this will be stored separately from your questionnaire and cannot be linked to your responses. Providing this information is voluntary.

Your participation is voluntary. Your data will only be used if you consent to participate. If you do not wish to participate, you will not face any disadvantages. You can withdraw at any time, even after giving consent and without providing reasons, by simply closing the window.

For the sake of readability, in some places, we have omitted the simultaneous use of male, female, and diverse gender forms (m/w/d). All references to individuals apply equally to all genders.

Project team:

Cheyenne Topf (Research Associate), Dr. Pola Hahlweg (Principal Investigator), Prof. Dr. Isabelle Scholl (Principal Investigator)

If you have read and understood the project and data protection information and wish to participate in the survey, please consent below. Please save this PDF so you can refer to your consent at any time. If you do not wish to participate, you do not need to take any action.

<u>Today's date:</u>
DD MM YYYY

1. Are you a patient with cancer?
☐ Yes ☐ No

Experiences with the provision of audio recordings of medical encounters
The following pages inquire about your experiences regarding the provision of audio recordings of medical encounters.

2. Have you ever had the experience of audio recording medical encounters?
☐ Yes ☐ No

3. How frequently have you made audio recordings of medical encounters?
☐ Once ☐ Multiple times

4. *[If experiences: "yes"]*
Who suggested making the audio recording? *(Multiple answers possible)*
☐ Myself ☐ My physician ☐ Another person *(please specify)*: ____________

5. *[If experiences: "yes"]*
How was the audio recording made? *(Multiple answers possible)*
☐ With an audio recording device ☐ with a mobile phone
☐ Other method *(please specify):* ______________________

6. Who made the audio recording? *(Multiple answers possible)*
☐ Myself ☐ My physician ☐ My accompanying person
☐ Another person *(please specify):* ______________________

7. *[If audio recording made by physician:]*
How was the audio recording provided to you? *(Multiple answers possible)*
☐ With a USB stick ☐ via Email ☐Over the internet (e.g. patient portal, cloud storage)
☐ Other method (please specify): ______________________

8. *[If experiences "yes":]*
Did you listen to the audio recording(s) again afterwards?
☐ Yes ☐ No

9. *[If listened: "yes":]*
With whom did you listen to the audio recording(s)? *(Multiple answers possible)*
☐ Alone ☐ With my partner ☐ With my family ☐ With friends ☐ With my physician

☐ With my caregiver ☐ With another person

10. Have you ever secretly recorded a medical encounter?
☐ Yes ☐ No ☐ No response

Attitudes towards the provision of audio recordings of medical encounters
The following questions concern your attitudes towards providing audio recordings of medical encounters. Please answer based on your current feelings. There are no right or wrong answers. Please try to provide your level of agreement spontaneously and generally.

11. How would you describe your attitude towards the provision of consultation recordings for patients?
☐ Very negative ☐ Mostly negative ☐ Somewhat negative ☐ Somewhat positive

☐ Mostly positive ☐ Very positive ☐ No response

Please check the extent to which you agree with the following statements. When the term „consultation recording“ is used, it refers to the audio recording of medical encounters.

		Completely disagree	Partially disagree	Slightly disagree	Slightly agree	Partially agree	Completely agree	No response
12.	A consultation recording enhances the understanding of information.	☐	☐	☐	☐	☐	☐	☐
13.	A consultation recording allows patients to retrospectively verify correct understanding of the information.	☐	☐	☐	☐	☐	☐	☐
14.	A consultation recording allows patients to ensure that the physician has understood them correctly.	☐	☐	☐	☐	☐	☐	☐
15.	A consultation recording allows patients to have a	☐	☐	☐	☐	☐	☐	☐

	better recall of the information discussed.							
16.	A consultation recording allows patients to prepare for follow-up appointments (.e.g. note down questions)	☐	☐	☐	☐	☐	☐	☐
17.	A consultation recording improves the quality of communication.	☐	☐	☐	☐	☐	☐	☐
18.	I am concerned that the quality of the communication decreases through a consultation recording.	☐	☐	☐	☐	☐	☐	☐
19.	A consultation recording allows physicians to be more responsive of concerns and needs of patients.	☐	☐	☐	☐	☐	☐	☐
20.	I am concerned that patients would be reserved and less open if consultations were recorded.	☐	☐	☐	☐	☐	☐	☐
21.	I am concerned that physicians would be reserved and less open if consultations were recorded.	☐	☐	☐	☐	☐	☐	☐
22.	A consultation recording leads to physicians taking their patients more seriously.	☐	☐	☐	☐	☐	☐	☐
23.	A consultation recording improves the trust between	☐	☐	☐	☐	☐	☐	☐

	patients and physicians.							
24.	I am concerned that the trust between patients and physicians would decrease if consultations were recorded.	☐	☐	☐	☐	☐	☐	☐
25.	I am concerned that the physician-patient-relationship would be more formal if consultations were recorded.	☐	☐	☐	☐	☐	☐	☐
26.	I am concerned that a consultation recording would put pressure on physicians.	☐	☐	☐	☐	☐	☐	☐
27.	A consultation recording facilitates an equal collaboration between patient and physician.	☐	☐	☐	☐	☐	☐	☐
28.	A consultation recording facilitates patients' active and self-responsible managing of their disease.	☐	☐	☐	☐	☐	☐	☐
29.	A consultation recording allows patients to compare their treatment options and make the best decision.	☐	☐	☐	☐	☐	☐	☐
30.	A consultation recording encourages patients to engage with their diagnosis.	☐	☐	☐	☐	☐	☐	☐

31.	I am concerned that a consultation recording puts too much responsibility on patients.	☐	☐	☐	☐	☐	☐	☐
32.	I am concerned that physicians would refer to the recording of the last consultation if any questions came up afterwards.	☐	☐	☐	☐	☐	☐	☐
33.	I am concerned that listening to the consultation recording would be a psychological burden for patients.	☐	☐	☐	☐	☐	☐	☐
34.	A consultation recording allows patients to share information with other health care professionals.	☐	☐	☐	☐	☐	☐	☐
35.	A consultation recording allows patients to share information with their relatives.	☐	☐	☐	☐	☐	☐	☐
36.	A consultation recording allows relatives to provide better support to the patient.	☐	☐	☐	☐	☐	☐	☐
37.	I am concerned that relatives could pressure patients into allowing them to listen to their consultation recording.	☐	☐	☐	☐	☐	☐	☐
38.	A consultation recording provides	☐	☐	☐	☐	☐	☐	☐

	evidence of what was said and done.							
39.	A consultation recording provides a protection for patients and physicians.	☐	☐	☐	☐	☐	☐	☐
40.	A consultation recording provides evidence in case of malpractice.	☐	☐	☐	☐	☐	☐	☐
41.	I am concerned that a consultation recording would be used as evidence against physicians.	☐	☐	☐	☐	☐	☐	☐
42.	I am concerned about confidentiality and data protection if consultations were recorded.	☐	☐	☐	☐	☐	☐	☐
43.	A consultation recording is helpful for treatment planning.	☐	☐	☐	☐	☐	☐	☐
44.	A consultation recording allows a better adherence to medical instructions.	☐	☐	☐	☐	☐	☐	☐
45.	I am concerned that the technical requirements for making consultation recordings don’t exist.	☐	☐	☐	☐	☐	☐	☐
46.	I am concerned that recording consultations is too complicated for physicians.	☐	☐	☐	☐	☐	☐	☐
47.	I am concerned that recording consultations is too complicated for patients.	☐	☐	☐	☐	☐	☐	☐

48.	I am concerned about patients perceiving a recording device as stressful during consultations.	☐	☐	☐	☐	☐	☐	☐
49.	A consultation recording is especially helpful for older people.	☐	☐	☐	☐	☐	☐	☐
50.	A consultation recording is especially helpful for people with language barriers.	☐	☐	☐	☐	☐	☐	☐
51.	A consultation recording is especially helpful for people with cognitive deficits.	☐	☐	☐	☐	☐	☐	☐
52.	A consultation recording is especially helpful when starting or changing a treatment.	☐	☐	☐	☐	☐	☐	☐
53.	A consultation recording is especially helpful when treatments are complex and extensive.	☐	☐	☐	☐	☐	☐	☐
54.	A consultation recording is especially helpful in consultations in which treatment decisions are made.	☐	☐	☐	☐	☐	☐	☐
55.	A consultation recording should also be conducted when the diagnosis is communicated during the consultation.	☐	☐	☐	☐	☐	☐	☐

56.	A consultation recording should be made even in brief consultations with little amount of new information.	☐	☐	☐	☐	☐	☐	☐

Desire for the provision of audio recordings of medical encounters
The following questions pertain to your potential preferences regarding the provision of audio recordings of your own medical encounters.

57. In the future, would you like to have audio recordings of your medical consultations?
☐ Yes ☐No ☐ Maybe

58. *[If future desire "yes"or "maybe":]*
Could you imagine making these audio recordings yourself with your mobile phone?
☐Yes ☐ No

59. *[If future desire "yes" or "maybe":]*
Would you be willing to listen to such a recording after the consultation?
☐Yes ☐ No ☐ Maybe

60. *[If listening "yes":]*
I want to listen to what my doctor said.
☐ does not apply at all ☐ mostly does not apply ☐ somewhat does not apply
☐ somewhat applies ☐ mostly applies ☐ fully applies ☐ no response

61. *[If listening "yes":]*
I want to listen to what I said.
☐ does not apply at all ☐ mostly does not apply ☐ somewhat does not apply
☐ somewhat applies ☐ mostly applies ☐ fully applies ☐ no response

62. *[If future desire "yes" or "maybe":]*
Would you be willing to share such an audio recording with your family members?
☐ Yes, to listen together ☐ Yes, my family members should listen to the recording without me ☐ No ☐ No response

Your desire for participation
63. Who, in your opinion, should make treatment decisions?
Please select the statement that most closely aligns with your attitude.

☐ I prefer to make the decisions about which treatment I will receive.
☐ I prefer to make the final decision about my treatment after seriously considering my physician’s opinion.
☐ I prefer that my physician and I share responsibility for deciding which treatment is best for me.
☐ I prefer that my physician makes the final decision about which treatment will be used, but seriously considers my opinion.
☐ I prefer to leave all my decision regarding treatment to my physician.

Questions about your health literacy
On a scale from very easy to very difficult, how easy would you say it is to …

		Very difficult	Fairly difficult	Fairly easy	Very easy	Don’t know
64.	…find information on treatments of illnesses that concern you?	☐	☐	☐	☐	☐
65.	…find out where to get professional help when you are ill (Doctor, Pharmacist, Psychologist)?	☐	☐	☐	☐	☐
66.	…understand what your doctor says to you?	☐	☐	☐	☐	☐
67.	…understand your doctor’s or pharmacist’s instruction on how to take a prescribed medicine?	☐	☐	☐	☐	☐
68.	…judge when you may need to get a second opinion from another doctor?	☐	☐	☐	☐	☐
69.	…use information the doctor gives you to make decisions about your illness?	☐	☐	☐	☐	☐
70.	…follow instructions from your doctor or pharmacist?	☐	☐	☐	☐	☐
71.	…find information on how to manage mental health problems like stress or depression?	☐	☐	☐	☐	☐
72.	…understand health warnings about behavior such as smoking, low physical activity and drinking too much?	☐	☐	☐	☐	☐
73.	…understand why you need health screenings (Cancer screenings, blood glucose testing, blood pressure)?	☐	☐	☐	☐	☐

74.	…judge if the information on health risks in the media is reliable (TV, Internet or other media)?	☐	☐	☐	☐	☐
75.	…decide how you can protect yourself from illness based on information in the media (Magazines, brochures, Internet or other media)?	☐	☐	☐	☐	☐
76.	…find out about activities that are good for your mental well-being (e.g. Meditation, physical exercise, walking, pilates)?	☐	☐	☐	☐	☐
77.	… understand advice on health from family members or friends?	☐	☐	☐	☐	☐
78.	…understand information in the media on how to get healthier (Internet, Magazines)?	☐	☐	☐	☐	☐
79.	…judge which everyday behaviour is related to your health (e.g. food and alcohol consumption, physical exercise)?	☐	☐	☐	☐	☐

Health literacy of chronically ill patients

How much difficulty do you have in talks with doctors, therapists or nursing staff…

		Very difficult	Fairly difficult	Fairly easy	Very easy	Don’t know
80.	…understanding medical information given to you	☐	☐	☐	☐	☐
81.	…applying the medical advice you have received at home on a daily basis	☐	☐	☐	☐	☐
82.	…understanding what the medical information means in terms of your disease	☐	☐	☐	☐	☐
83.	…making it clear to doctors, therapists and nursing staff how important your questions are for you	☐	☐	☐	☐	☐
84.	…understanding the wealth of information conveyed	☐	☐	☐	☐	☐

85.	…communicating what you already know and don't know about your disease to doctors, therapists and nursing staff	☐	☐	☐	☐	☐
86.	…understanding foreign words	☐	☐	☐	☐	☐
87.	…approaching staff when a problem of yours has not been adequately addressed	☐	☐	☐	☐	☐
88.	…distinguishing essential from less important information	☐	☐	☐	☐	☐
89.	…also understanding difficult medical information	☐	☐	☐	☐	☐
90.	…addressing your own problems and issues	☐	☐	☐	☐	☐
91.	…understanding medical terminology	☐	☐	☐	☐	☐
92.	…talking about your questions	☐	☐	☐	☐	☐
93.	…absorbing the amount of new information	☐	☐	☐	☐	☐
94.	…communicating your own expectations and wishes in terms of your therapy	☐	☐	☐	☐	☐
95.	…understanding complex sentences	☐	☐	☐	☐	☐
96.	…posing very personal questions about your disease	☐	☐	☐	☐	☐
97.	…understanding medical information immediately	☐	☐	☐	☐	☐

Affinity for Technology Interaction

In the following questionnaire, we will ask you about your interaction with technical systems. The term "technical systems" refers to apps and other software applications, as well as entire digital devices (e.g., mobile phone, computer, TV, car navigation).

Please indicate the degree to which you agree/disagree with the following statements.

		Completely disagree	Largely disagree	Slightly disagree	Slightly agree	Completely agree	Completely agree

98.	I like to occupy myself in greater detail with technical systems.	☐	☐	☐	☐	☐	☐
99.	I like testing the functions of new technical systems	☐	☐	☐	☐	☐	☐
100.	It is enough for me that a technical system works; I don't care how or why.	☐	☐	☐	☐	☐	☐
101.	It is enough for me to know the basic functions of a technical system.	☐	☐	☐	☐	☐	☐

Knowledge about law

102. Do you know the laws that regulate audio recordings of conversations in Germany?
☐ Yes ☐ No ☐ No response

Questions about your person
Please provide some information about yourself below.

103. Age in years:
☐ Younger than 18 years ☐ 18-29 years ☐ 30-39 years ☐ 40-49 years
☐ 50-59 years ☐ 60-69 years ☐ 70-79 years ☐ 80 years and older

104. Gender:
☐ Female ☐ Male ☐ Non-binary/diverse

105. How would you rate your knowledge of the German language?

	1	2	3	4	5	6	7	8	9	10	
Very poor	☐	☐	☐	☐	☐	☐	☐	☐	☐	☐	Very good

106. Highest (Educational) Degree:
☐ No formal education
☐ Lower Secondary Education
☐ Intermediate Secondary Education
☐ High School Diploma/General Certificate of Education
☐ Technical/Vocational School Diploma

☐ College/University Degree
☐ Other Degree

107. In which federal state do you live?
☐ Schleswig-Holstein
☐ Hamburg
☐ Lower Saxony
☐ Bremen
☐ North Rhine-Westphalia
☐ Hesse
☐ Rhineland-Palatinate
☐ Baden-Württemberg
☐ Bavaria
☐ Saarland
☐ Berlin
☐ Brandenburg
☐ Mecklenburg-Western Pomerania
☐ Saxony
☐ Saxony-Anhalt
☐ Thuringia

Questions about your illness and medical encounters:

108. Which of the following cancer types have you been diagnosed with? (*Multiple answers possible)*
☐ Breast cancer
☐ Prostate cancer
☐ Colorectal cancer
☐ Lung cancer
☐ Skin cancer
☐ Lymphoma
☐ Ovarian cancer
☐ Oral cavity and oropharyngeal cancer
☐ Leukemia
☐ Pancreatic cancer
☐ Kidney cancer
☐ Thyroid cancer
☐ Cervical cancer
☐ Liver cancer
☐ Esophageal cancer
☐ Other cancer types
☐ No response

109. When was your initial diagnosis?

☐ Less than 1 year ago ☐ 1 to 5 years ago ☐ More than 5 years ago
☐ No response

110. What is the current status of disease progression?
☐ Localized ☐ Metastasized ☐ In remission/"cured" ☐ Other status
☐ No response

111. How would you describe current state of health?
☐ Bad ☐ Less Good ☐ Good ☐ Very good ☐ Excellent ☐ No Response

112. How many consultations regarding your cancer have you had in the last 6 months?
☐ Less than 3 ☐ 3-5 ☐ More than 5 ☐ No response
