# Supplementary material for: Attitudes and experiences of cancer patients toward the provision of audio recordings of their own medical encounter: a cross-sectional online survey
Source: Front Psychol. 2024 Jun 19;15:1378854. doi: 10.3389/fpsyg.2024.1378854 (PMC11220273; doi:10.3389/fpsyg.2024.1378854)
Supplement: Supplementary file 4 [file Data_Sheet_4.PDF]

SUPPLEMENTARY FILE 4

Table

Distribution of invitation for online survey

Way of distribution	N
**Contacted by email:** Number of groups contacted…	
Self-help groups[a] (nationwide)	1047
**Distribution of invitation by email:** Number of invitations sent to…	
Newsletter for self-help groups and patient organizations of UKE[b]	1
„Haus der Krebs-Selbsthilfe - Bundesverband e.V.“ (Self-help association)[c]	1
**Posts on social media / websites:** Number of posts on…	
Instagram accounts	14
Self-help group websites	2
Self-help group Facebook pages	2
Social media accounts of UKE[b] (Instagram, Twitter)	2
Website of the outpatient clinic for psycho-oncology of UKE[b]	1
**Distribution of leaflets:** Number of leaflets sent to…	
Oncological in- and outpatient clinics (nationwide)	360
Outpatient clinic for psycho-oncology of UKE	180
Rehabilitation clinics (nationwide)	170
Gynecological outpatient clinic of UKE	100
20th Annual Congress of the Psycho-Oncology Working Group of the German Cancer Society	40
Hamburg Cancer Society	40
Psychotherapeutic offices (Hamburg)	35
Symposium of the “Gesundheitskiosk Billstedt-Horn”	10
Cancer counselling center (Hamburg/Pinneberg)	10

Notes: [a] for various types of cancer; [b] UKE = University Medical Center Hamburg-Eppendorf; [c] Invitation was forwarded to their affiliated associations.
