# Supplementary material for: Attitudes and experiences of cancer patients toward the provision of audio recordings of their own medical encounter: a cross-sectional online survey
Source: Front Psychol. 2024 Jun 19;15:1378854. doi: 10.3389/fpsyg.2024.1378854 (PMC11220273; doi:10.3389/fpsyg.2024.1378854)

SUPPLEMENTARY FILE 5

Frequency distributions of the statements about benefits of consultation recordings, ranging from completely disagree (=1) to completely agree (=6).

Ordered from highest to lowest mean.

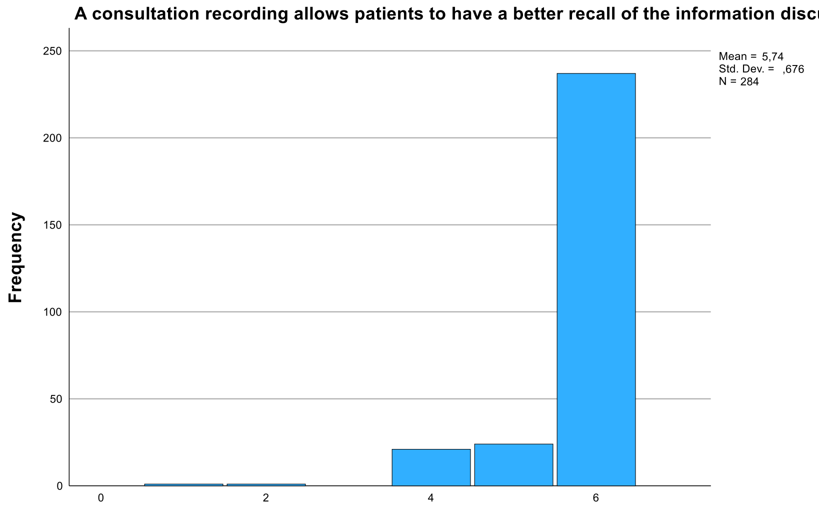

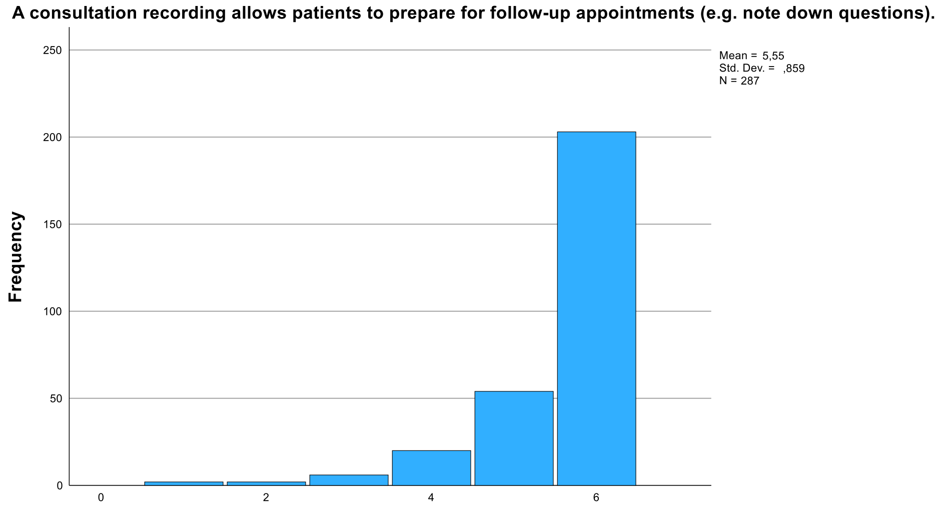

A consultation recording allows patients to retrospectively verify correct understanding of the information

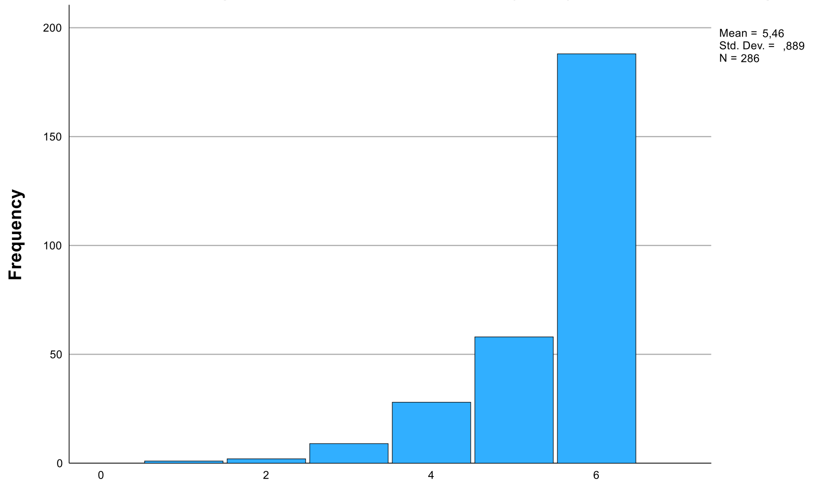

A consultation recording enhances the understanding of information.

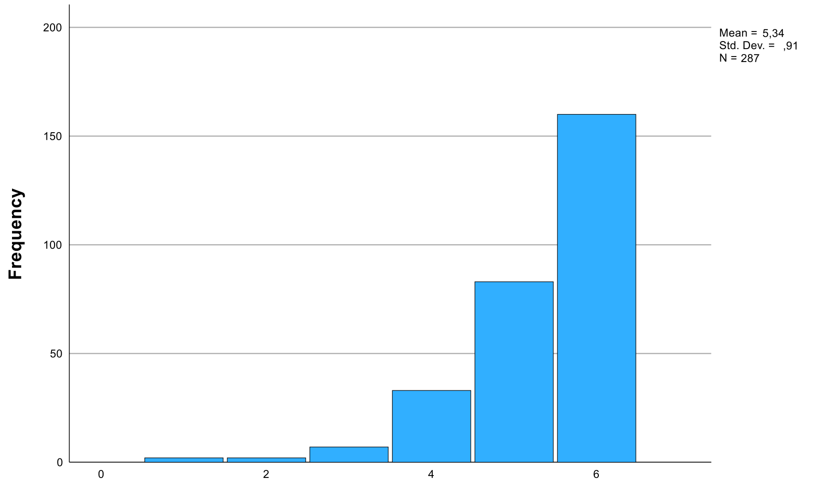

A consultation recording is especially helpful in consultations in which treatment decisions are made.

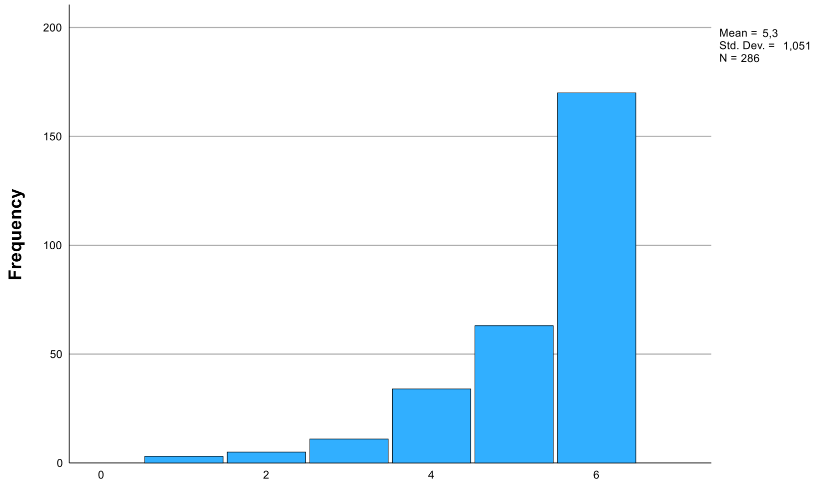

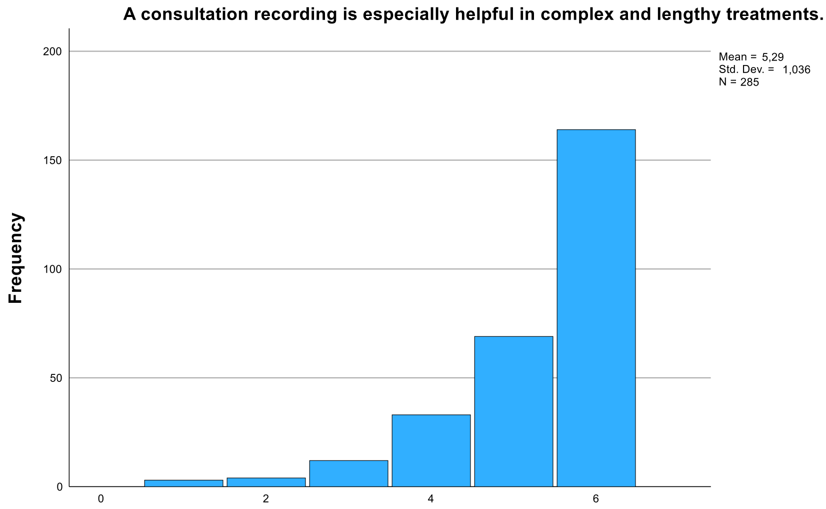
A consultation recording is especially helpful in complex and lengthy treatments.
Frequency
Mean = 5,29
Std. Dev. = 1,036
N = 285
0
50
100
150
200
0
2
4
6

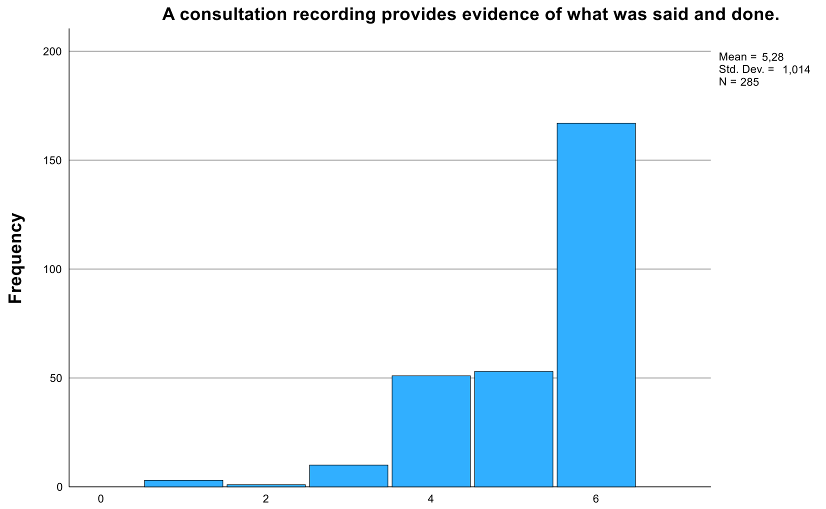
A consultation recording provides evidence of what was said and done.
Frequency
Mean = 5,28
Std. Dev. = 1,014
N = 285
0
50
100
150
200
0
2
4
6

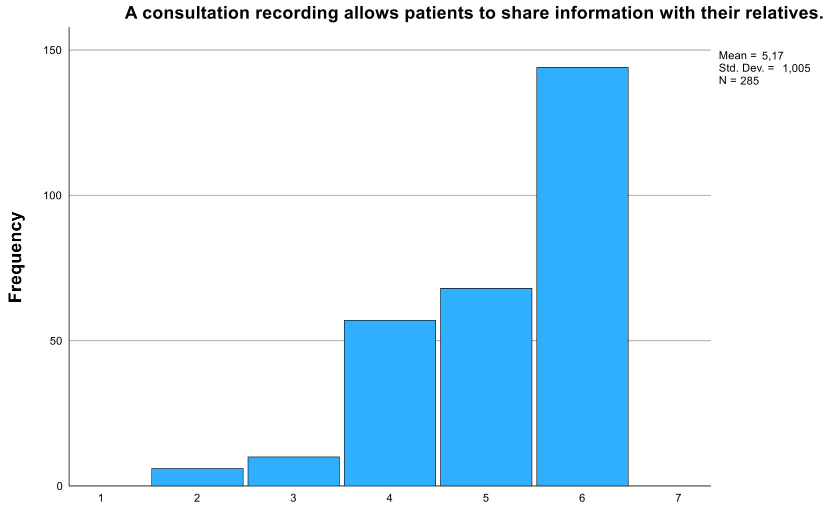
A consultation recording allows patients to share information with their relatives.
Frequency
Mean = 5,17
Std. Dev. = 1,005
N = 285
0
50
100
150
1
2
3
4
5
6
7

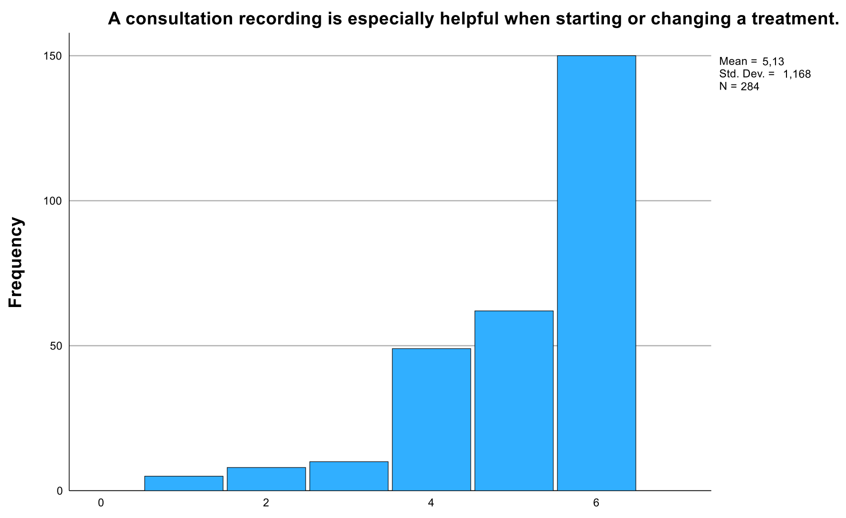
A consultation recording is especially helpful when starting or changing a treatment.
Frequency
Mean = 5,13
Std. Dev. = 1,168
N = 284
0
50
100
150
0
2
4
6

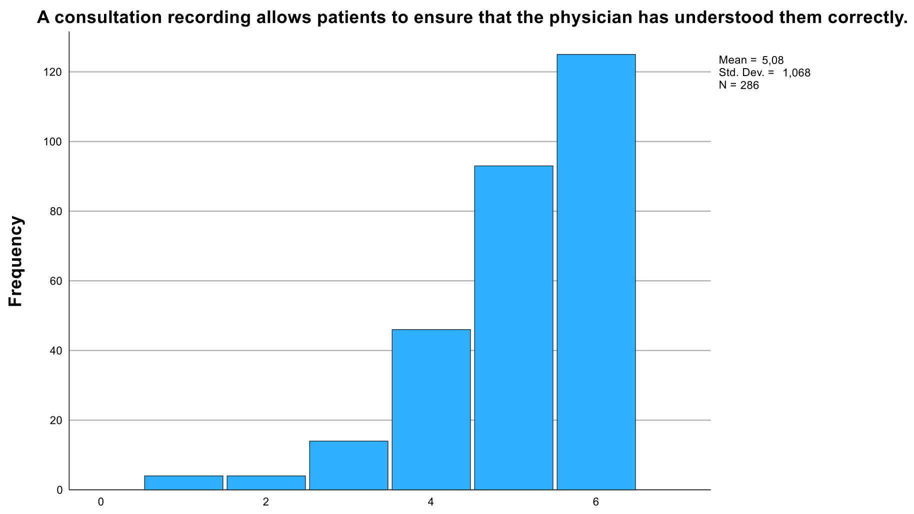
A consultation recording allows patients to ensure that the physician has understood them correctly.
Frequency
Mean = 5,08
Std. Dev. = 1,068
N = 286
0
20
40
60
80
100
120
0
2
4
6

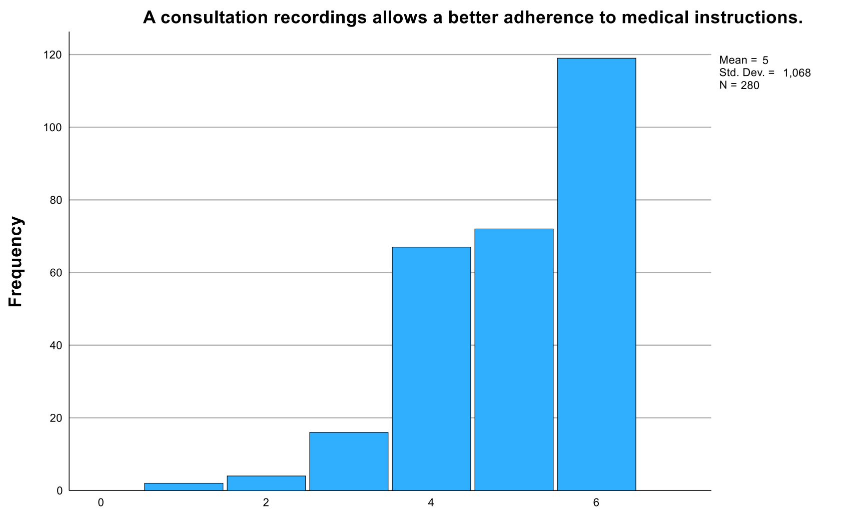
A consultation recordings allows a better adherence to medical instructions.
Frequency
Mean = 5
Std. Dev. = 1,068
N = 280
0
20
40
60
80
100
120
0
2
4
6

A consultation recording is especially helpful for people with language barriers.

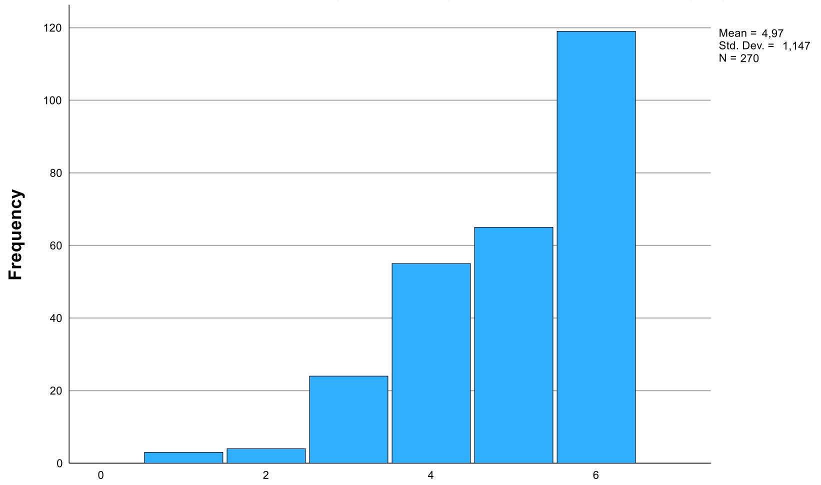

A consultation recording allows patients to compare their treatment options and make the best decision.

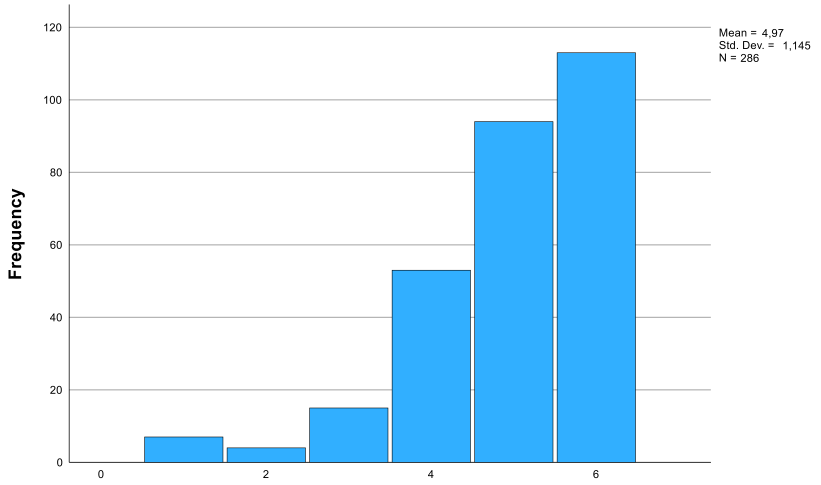

A consultation recording should also be conducted when the diagnosis is communicated during the consultation.

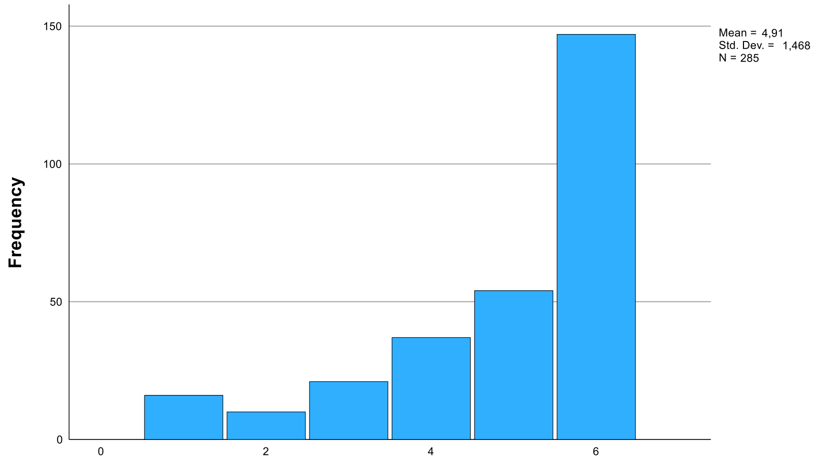

A consultation recording is especially helpful for people with cognitive deficits.

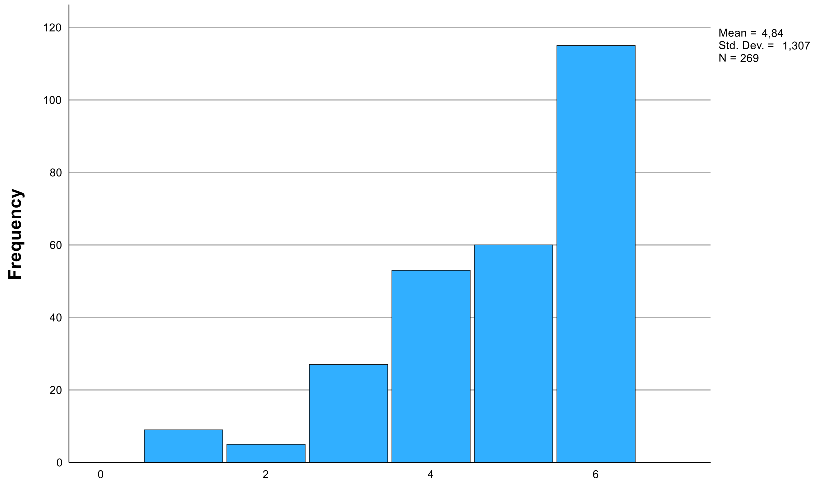

A consultation recording is especially helpful for older people.

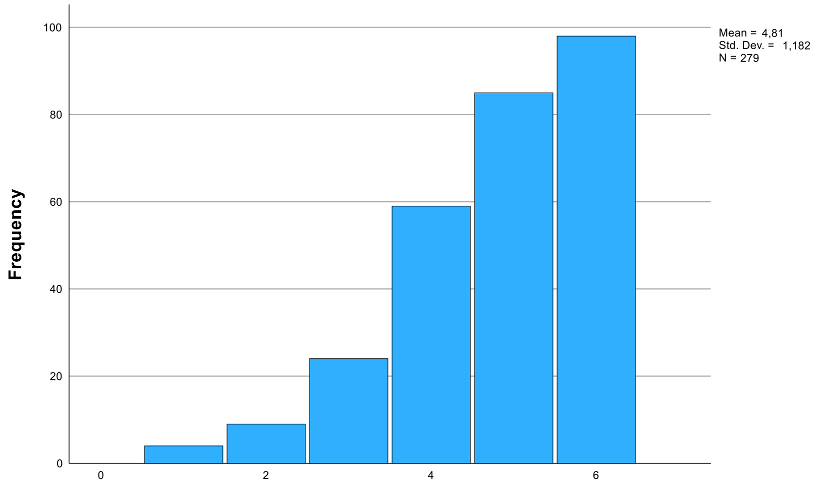

A consultation recordings allows relatives to provide better support to the patient.

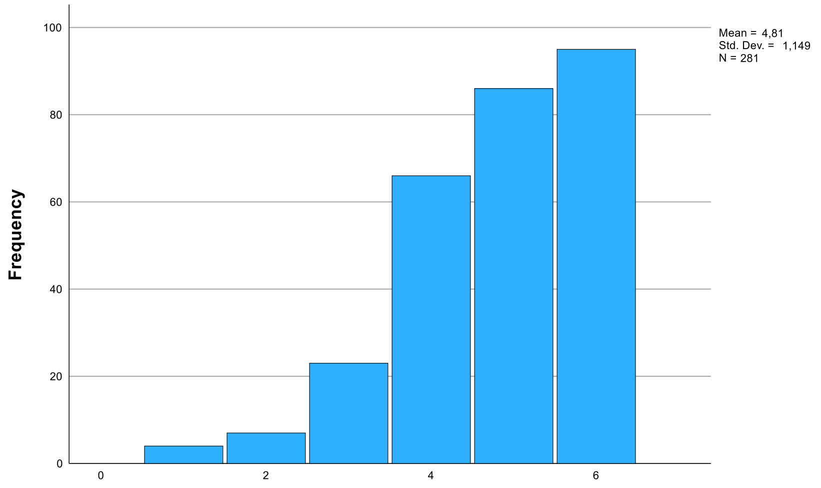

A consultation recording allows patients to share information with other healthcare professionals.

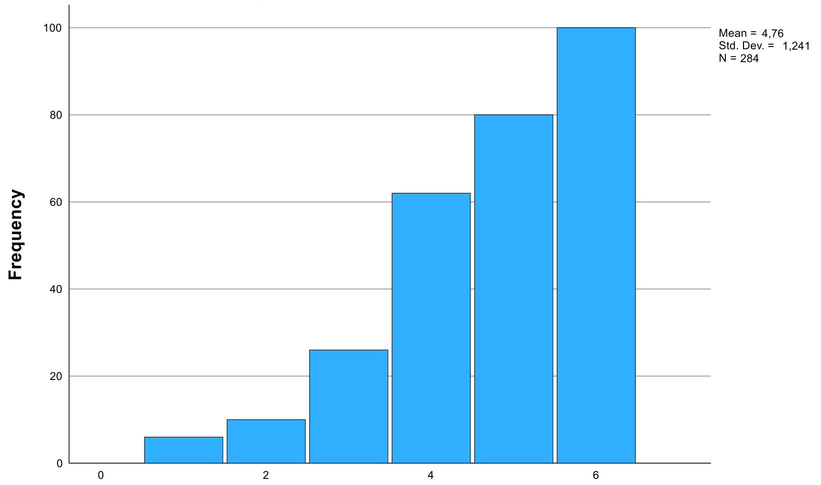

A consultation recording encourages patients to engage with their diagnosis.

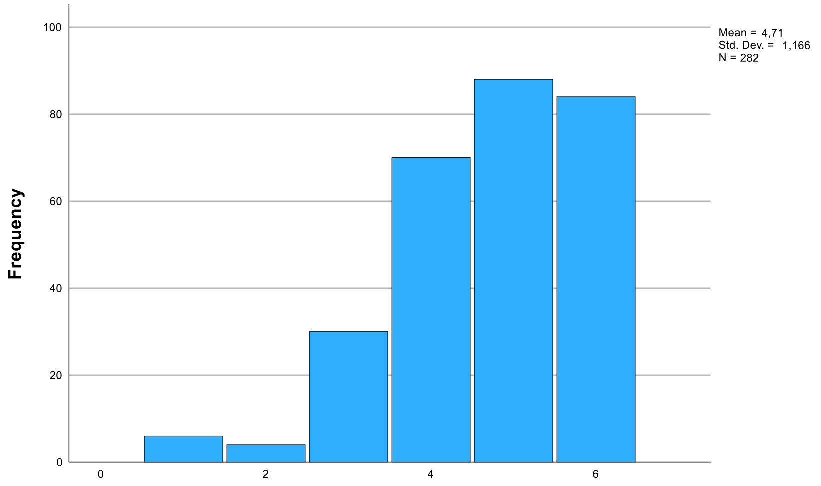

A consultation recording is helpful for treatment planning.

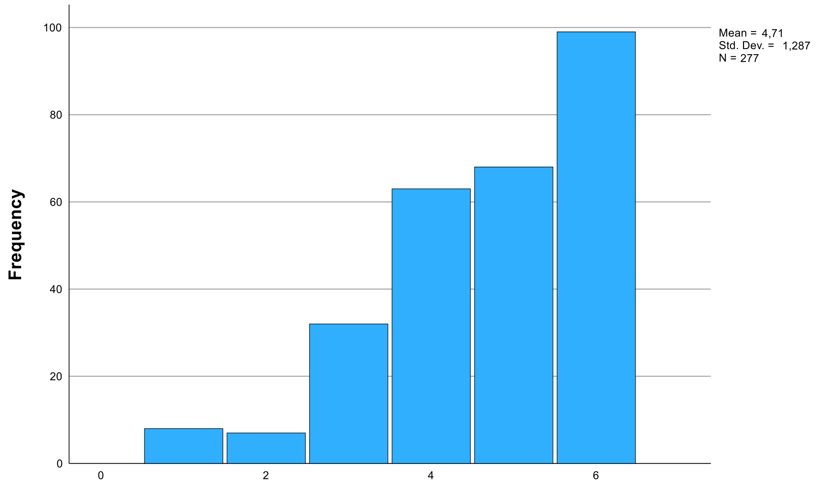

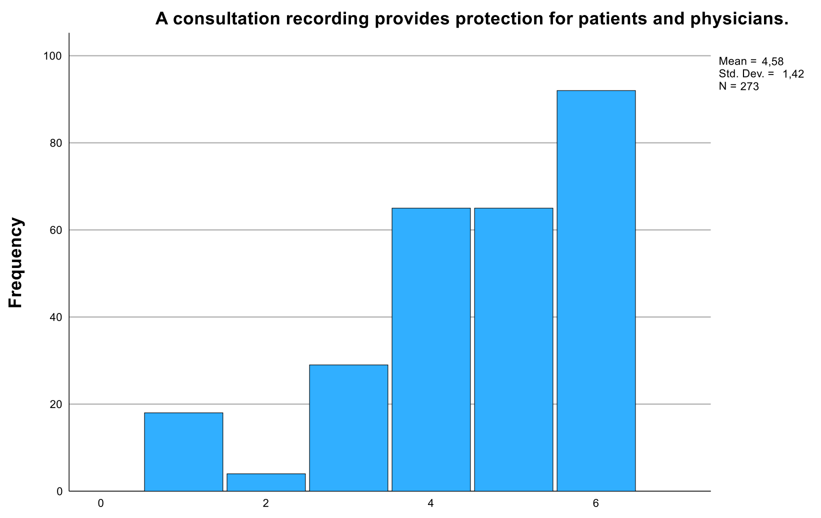
A consultation recording provides protection for patients and physicians.
Mean = 4,58
Std. Dev. = 1,42
N = 273
Frequency
100
80
60
40
20
0
0
2
4
6

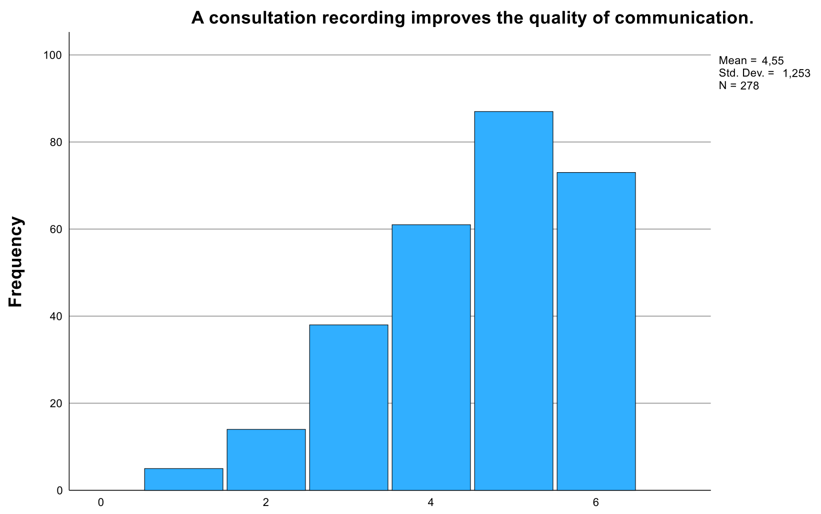
A consultation recording improves the quality of communication.
Mean = 4,55
Std. Dev. = 1,253
N = 278
Frequency
100
80
60
40
20
0
0
2
4
6

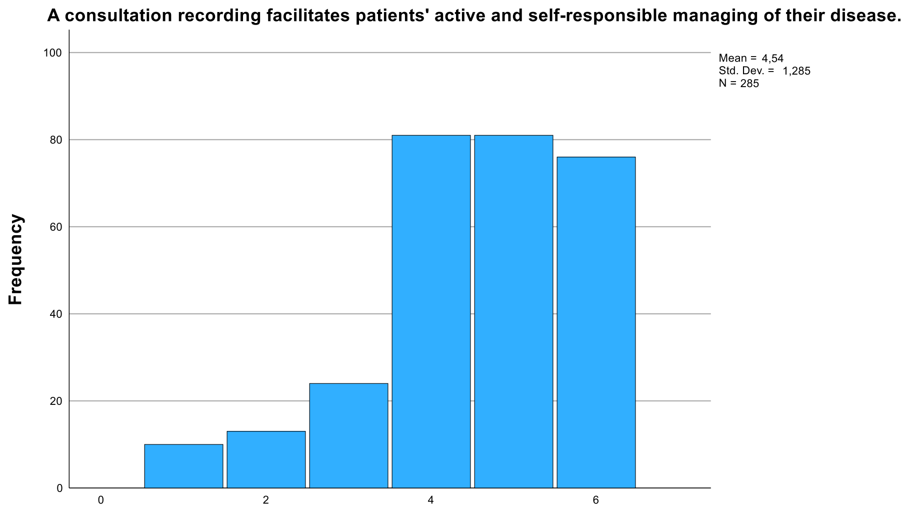
A consultation recording facilitates patients' active and self-responsible managing of their disease.
Mean = 4,54
Std. Dev. = 1,285
N = 285
Frequency
100
80
60
40
20
0
0
2
4
6

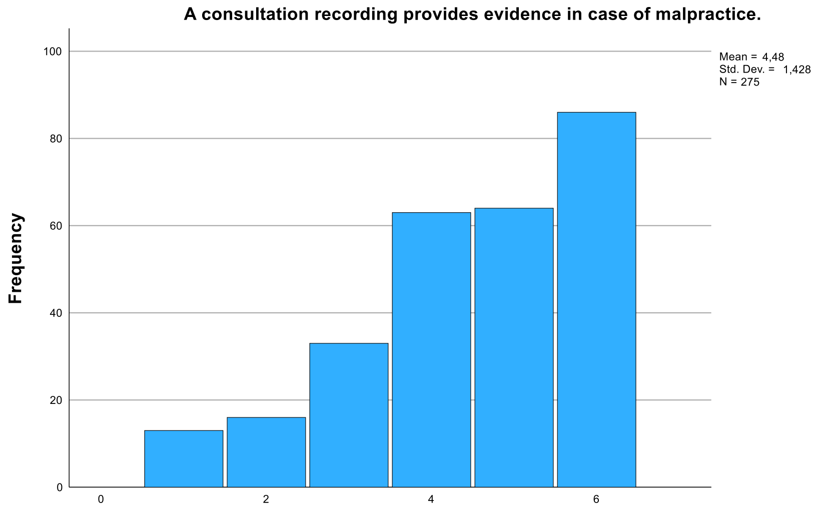
A consultation recording provides evidence in case of malpractice.
Mean = 4,48
Std. Dev. = 1,428
N = 275
Frequency
0
20
40
60
80
100
0
2
4
6

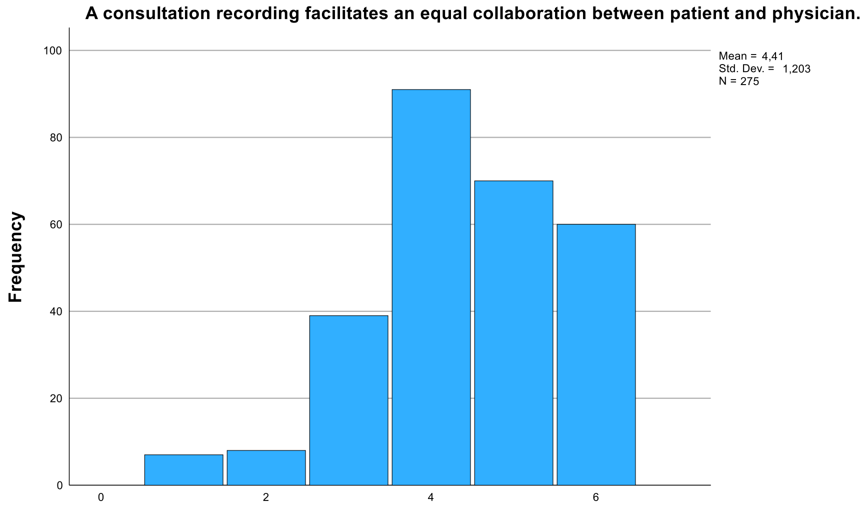
A consultation recording facilitates an equal collaboration between patient and physician.
Mean = 4,41
Std. Dev. = 1,203
N = 275
Frequency
0
20
40
60
80
100
0
2
4
6

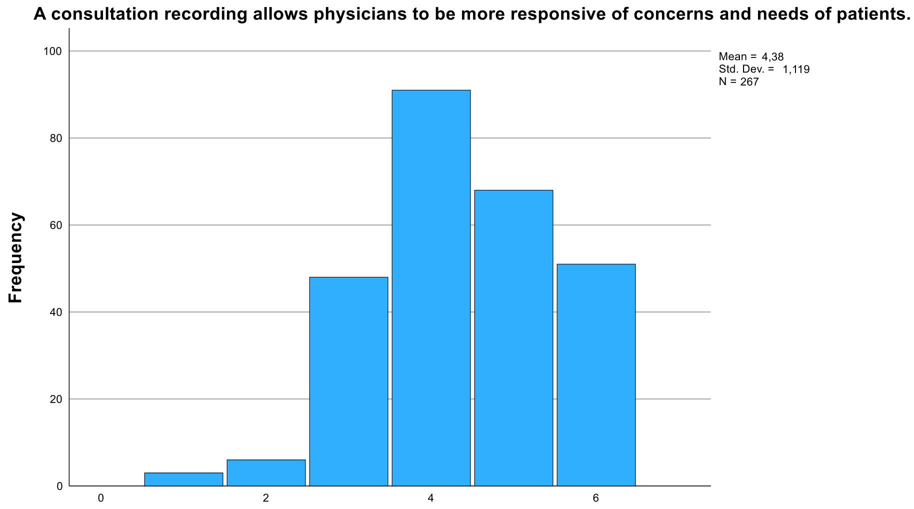
A consultation recording allows physicians to be more responsive of concerns and needs of patients.
Mean = 4,38
Std. Dev. = 1,119
N = 267
Frequency
0
20
40
60
80
100
0
2
4
6

A consultation recording leads to physicians taking their patients more seriously.

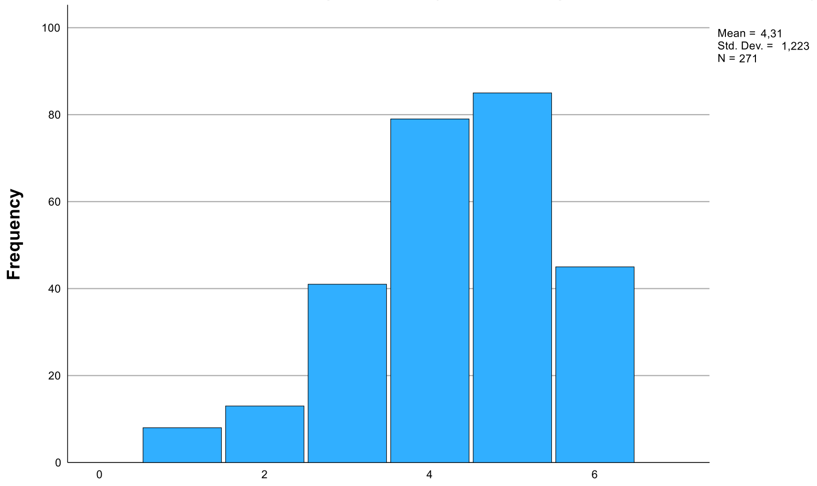

A consultation recording should be made even in brief consultations with little amount of new information.

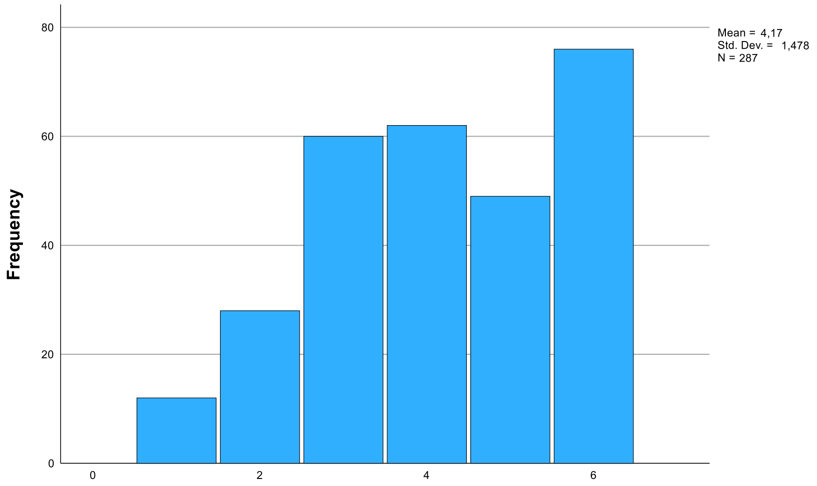

A consultation recording improves the trust between patients and physicians.

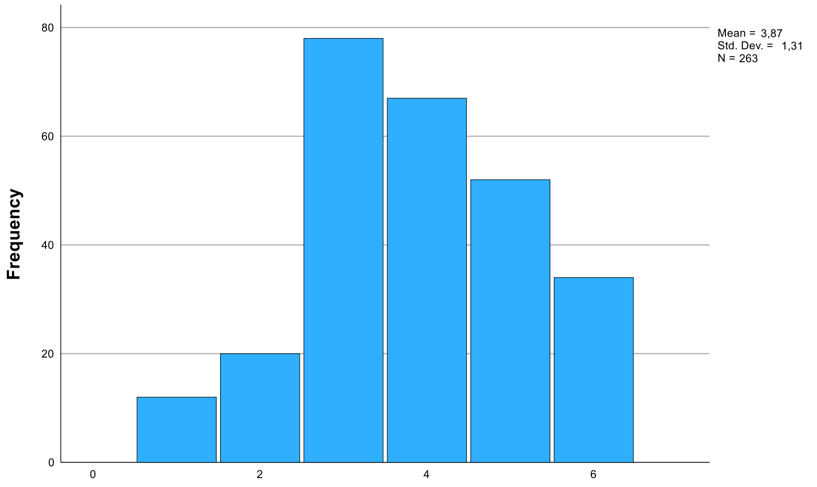

Supplement: Supplementary file 5 [file Data_Sheet_5.PDF]
