# Supplementary material for: Attitudes and experiences of cancer patients toward the provision of audio recordings of their own medical encounter: a cross-sectional online survey
Source: Front Psychol. 2024 Jun 19;15:1378854. doi: 10.3389/fpsyg.2024.1378854 (PMC11220273; doi:10.3389/fpsyg.2024.1378854)
Supplement: Supplementary file 6 [file Data_Sheet_6.PDF]

SUPPLEMENTARY FILE 6

Frequency distributions of the statements about concerns of consultation recordings, ranging from completely disagree (=1) to completely agree (=6).

Ordered from highest to lowest mean.

I am concerned that a consultation recording would put pressure on physicians.

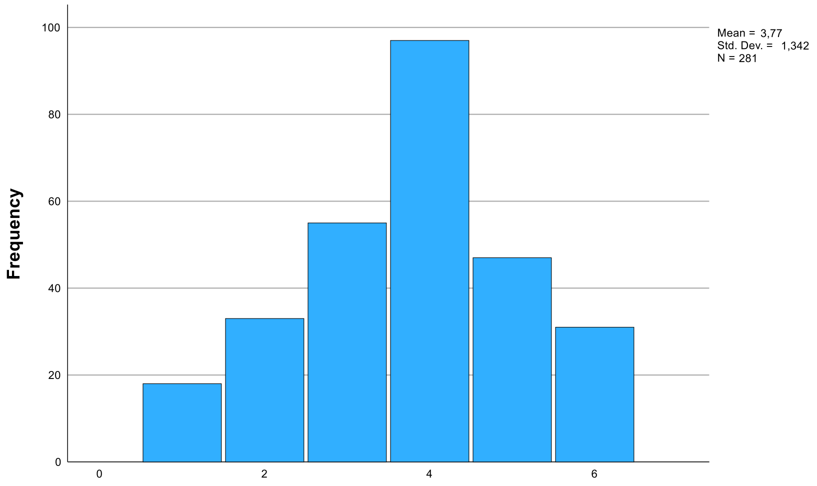

I am concerned that physicians would be reserved and less open if consultations were recorded.

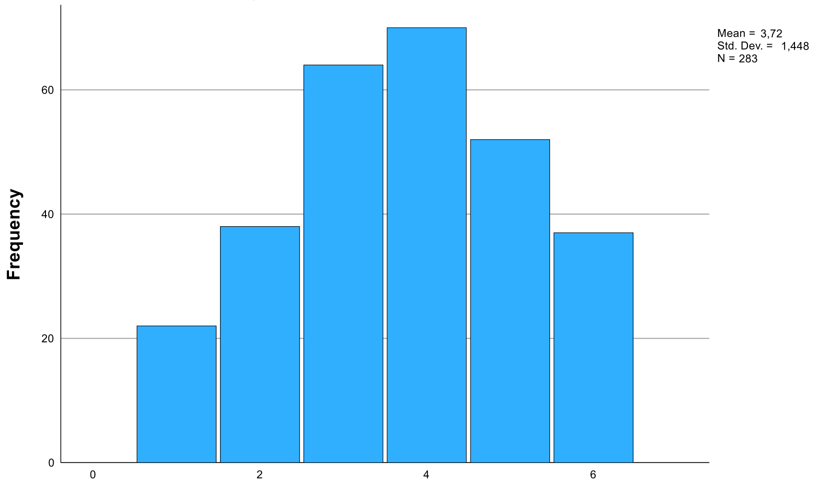

I am concerned that the physician-patient relationship would be more formal if consultations were recorded.

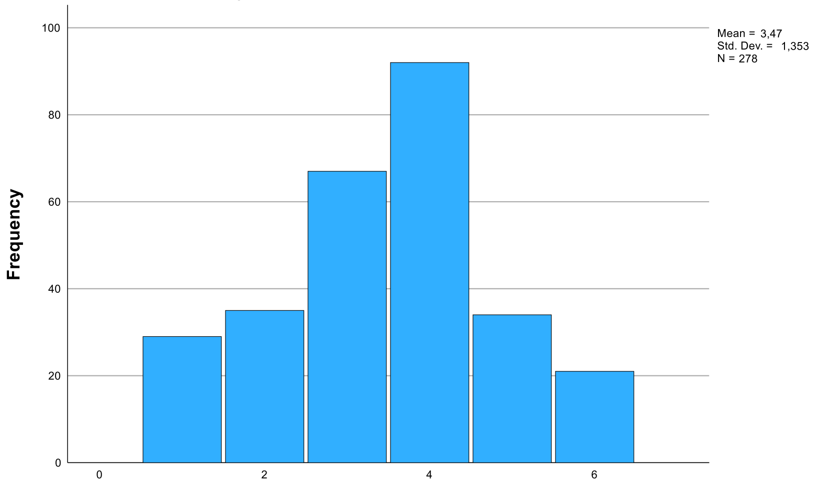

I am concerned that physicians would refer to the recording of the last consultation if any questions came up afterwards.

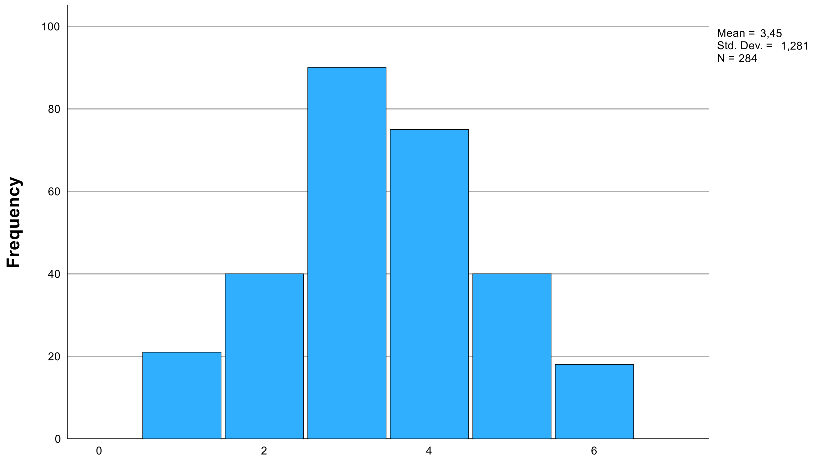

I am concerned that a consultation recording would be used as evidence against physicians.

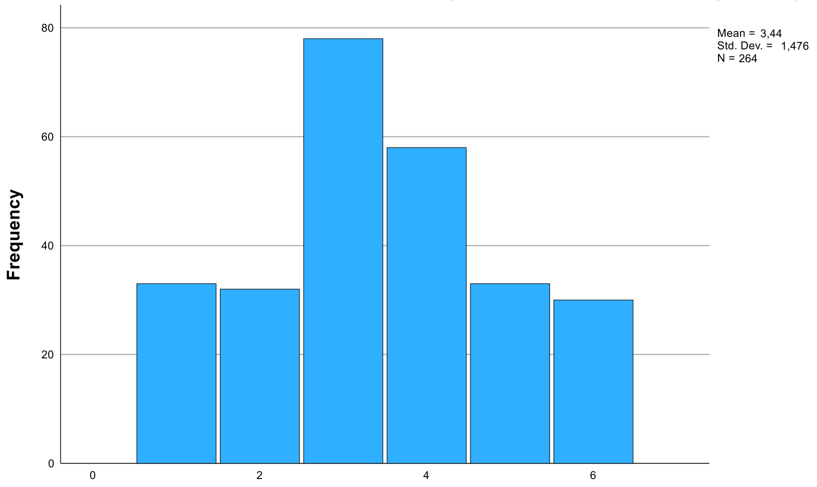

I am concerned that relatives could pressure patients into allowing them to listen to their consultation recording.

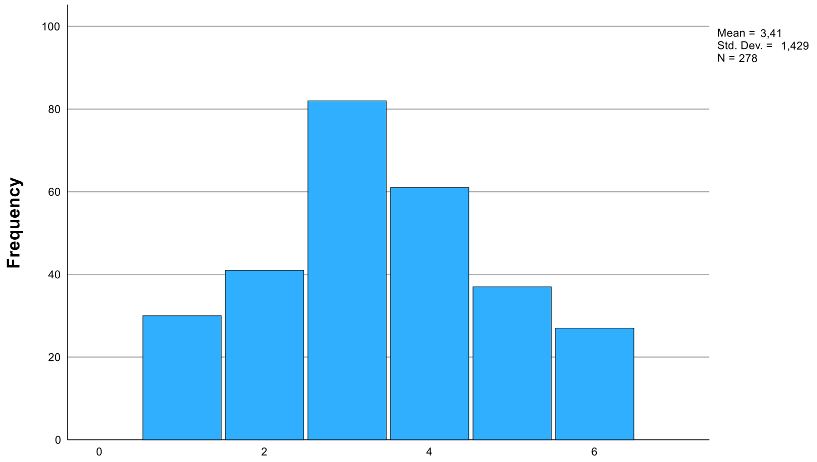

I am concerned about confidentiality and the data protection if consultations were recorded.

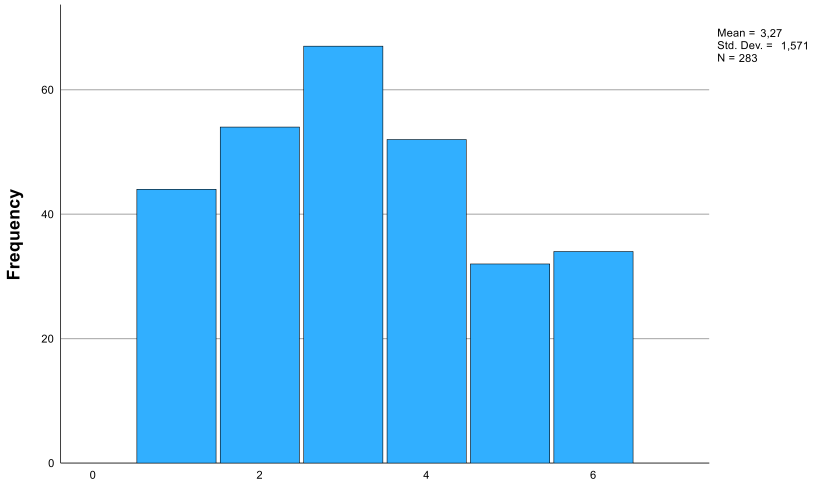

I am concerned that listening to the consultation recording would be a psychological burden for patients.

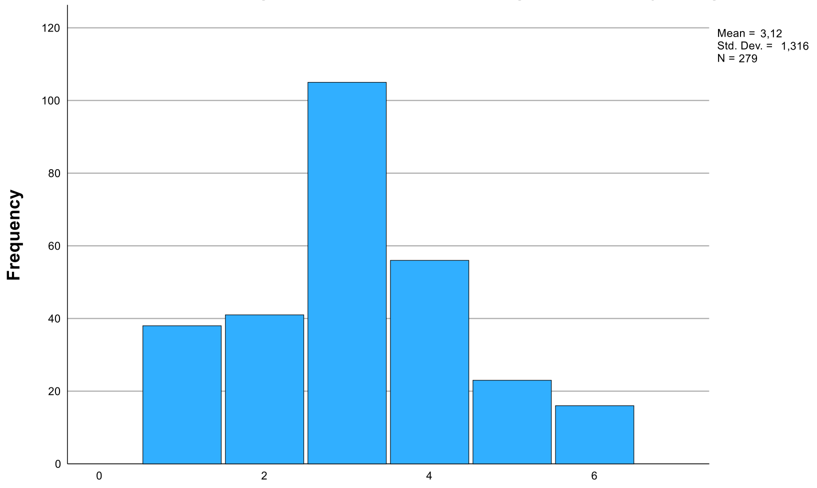

I am concerned about patients perceiving a recording device as stressful during consultations.

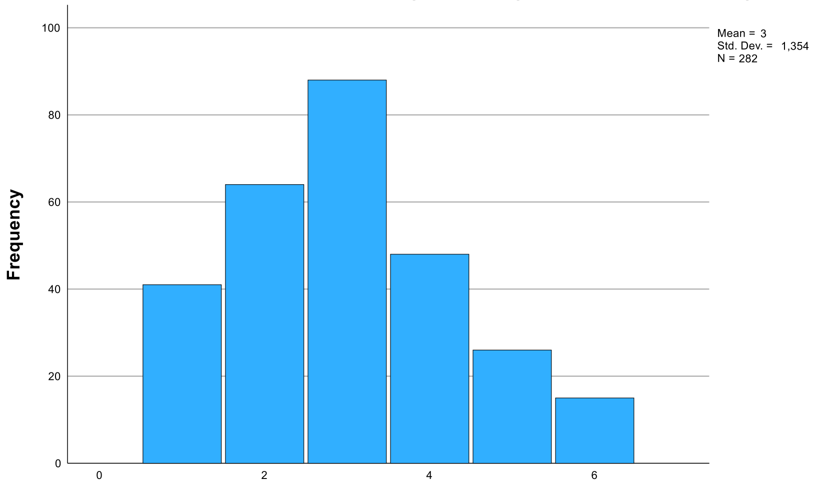

I am concerned that patients would be reserved and less open if consultations were recorded.

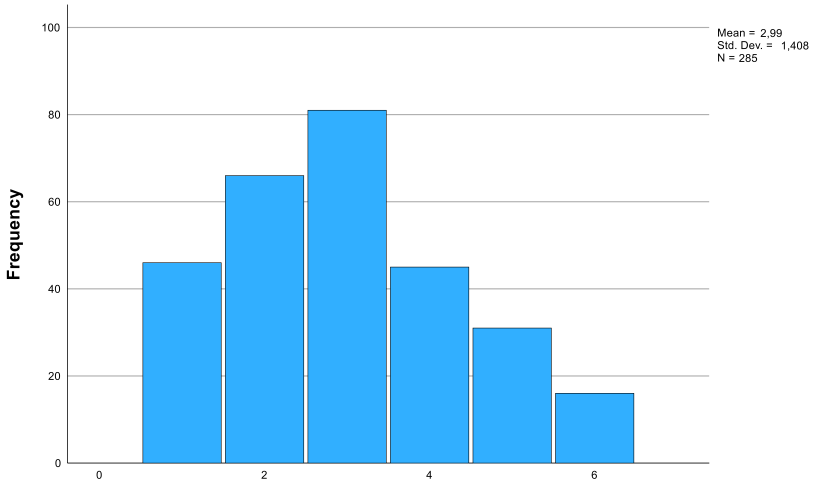

I am concerned that the technical requirements for making consultation recordings do not exist.

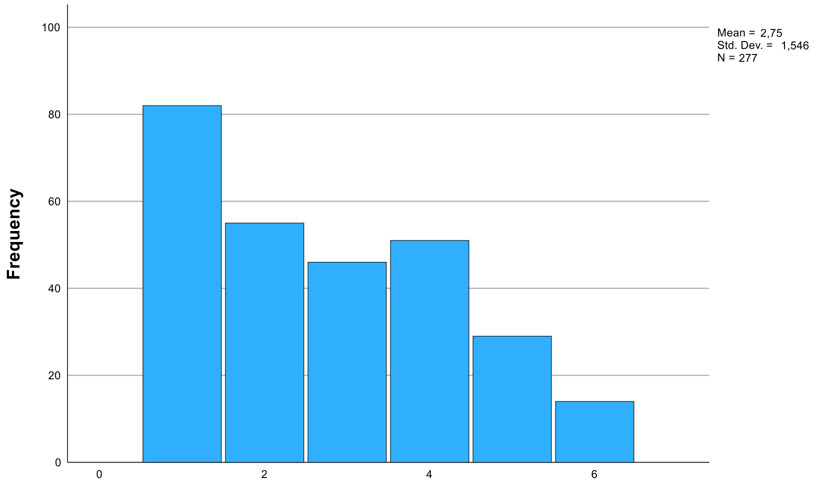

I am concerned that the trust between patients and physicians would decrease if consultations were recorded.

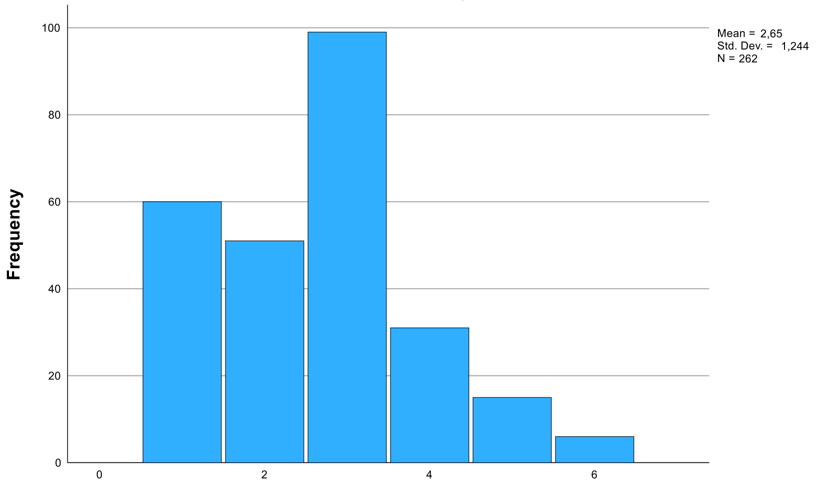

I am concerned that a consultation recording puts too much responsibility on patients.

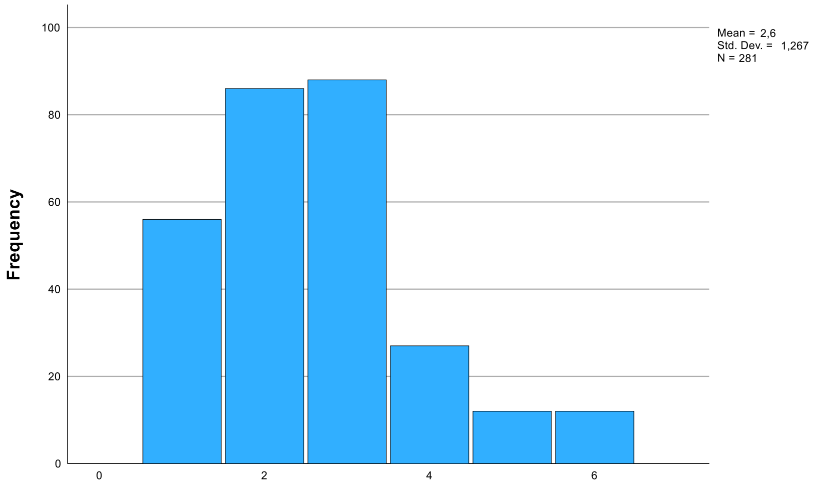

I am concerned that recording consultations is too complicated for physicians.

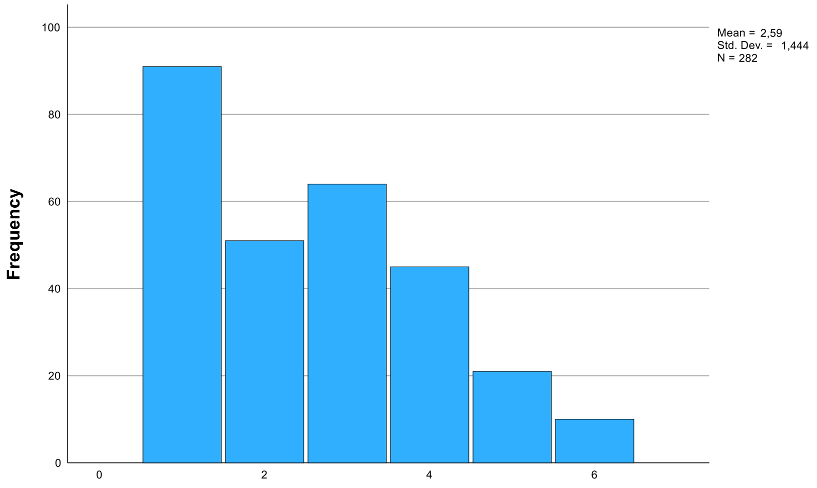

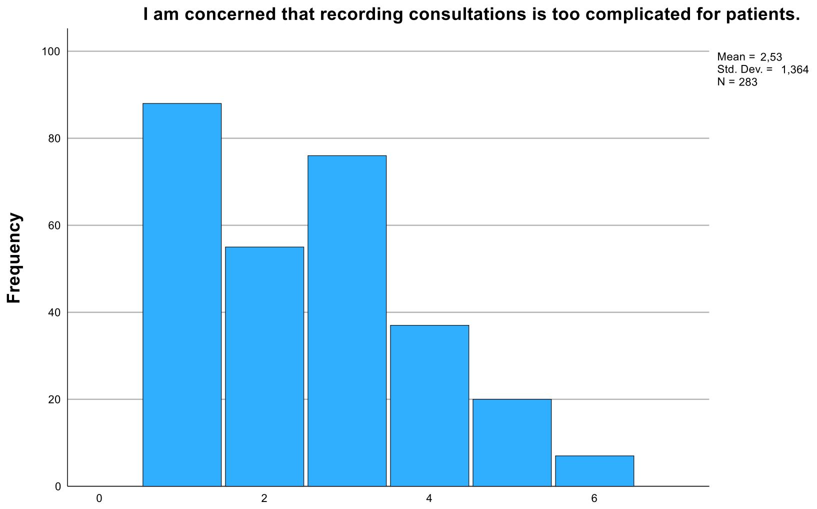
I am concerned that recording consultations is too complicated for patients.
Frequency
100
80
60
40
20
0
0
2
4
6
Mean = 2,53
Std. Dev. = 1,364
N = 283

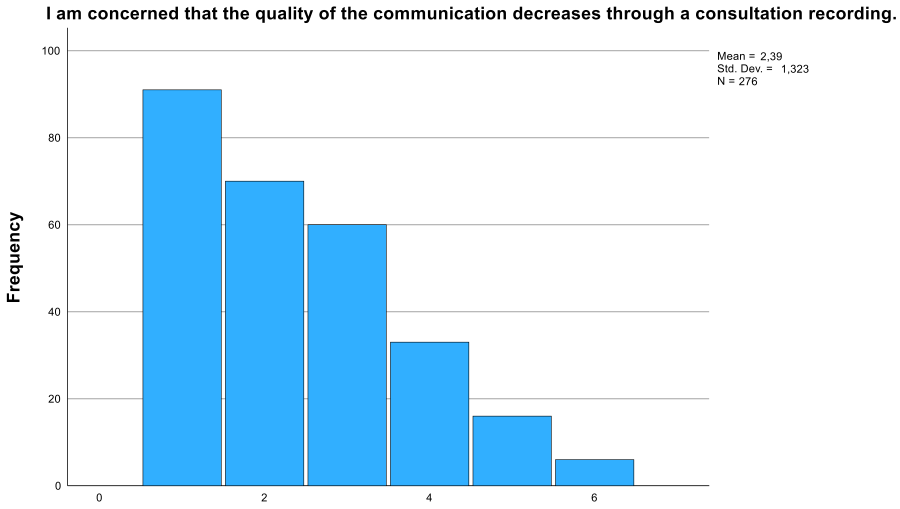
I am concerned that the quality of the communication decreases through a consultation recording.
Frequency
100
80
60
40
20
0
0
2
4
6
Mean = 2,39
Std. Dev. = 1,323
N = 276
