# Supplementary material for: Attitudes and experiences of cancer patients toward the provision of audio recordings of their own medical encounter: a cross-sectional online survey
Source: Front Psychol. 2024 Jun 19;15:1378854. doi: 10.3389/fpsyg.2024.1378854 (PMC11220273; doi:10.3389/fpsyg.2024.1378854)
Supplement: Supplementary file 7 [file Data_Sheet_7.PDF]

SUPPLEMENTARY FILE 7

Attitudes towards consultation recordings within the complete sample and three sub samples (desire for consultation recordings in the future = yes/maye/no)

	**Complete Sample**			**Subsample: Desire for Consultation Recordings = Yes**			**Subsample: Desire for Consultation Recordings = Maybe**			**Subsample: Desire for Consultation Recordings = No**		
Item Desription	n	Mean (SD)	Median	n	Mean (SD)	Median	n	Mean (SD)	Median	n	Mean (SD)	Median
**General attitude towards consultation recording**												
General attitude towards consultation recordings for patients[1]	*283*	*5.22 (.99)*	*6.00*	*191*	*5.57 (.66)*	*6.00*	*72*	*4.75 (1.00)*	*5.00*	*20*	*3.60 (1.31)*	*3.00*
**Levels of agreement towards different statements about benefits of consultation recordings** A consultation recording…[2,3]												
…allows patients to have a better recall of the information discussed.	*284*	*5.74 (.68)*	*6.00*	*191*	*5.88 (.41)*	*6.00*	*73*	*5.66 (.65)*	*6.00*	*20*	*4.65 (1.24)*	*4.00*
…allows patients to prepare for follow-up appointments (e.g. note down questions).	*287*	*5.55 (.86)*	*6.00*	*193*	*5.68 (.76)*	*6.00*	*73*	*5.48 (.71)*	*6.00*	*21*	*4.52 (1.36)*	*5.00*
…allows patients to retrospectively verify correct understanding of the information.	*286*	*5.46 (.89)*	*6.00*	*192*	*5.68 (.69)*	*6.00*	*73*	*5.25 (.88)*	*5.00*	*21*	*4.24 (1.34)*	*4.00*
…enhances the understanding of information.	*287*	*5.34 (.91)*	*6.00*	*193*	*5.61 (.64)*	*6.00*	*73*	*5.12 (.76)*	*5.00*	*21*	*3.67 (1.43)*	*4.00*
…is especially helpful in consultations in which treatment decisions are made.	*286*	*5.30 (1.05)*	*6.00*	*192*	*5.58 (.81)*	*6.00*	*73*	*5.05 (.88)*	*5.00*	*21*	*3.67 (1.68)*	*4.00*
…is especially helpful in complex and lengthy treatments.	*285*	*5.29 (1.04)*	*6.00*	*192*	*5.60 (.73)*	*6.00*	*72*	*5.00 (.90)*	*5.00*	*21*	*3.48 (1.60)*	*4.00*
…provides evidence of what was said and done.	*285*	*5.28 (1.01)*	*6.00*	*192*	*5.48 (.93)*	*6.00*	*72*	*5.07 (.91)*	*5.00*	*21*	*4.19 (1.25)*	*4.00*
…allows patients to share information with their relatives.	*285*	*5.17 (1.00)*	*6.00*	*192*	*5.41 (.86)*	*6.00*	*72*	*4.76 (1.01)*	*5.00*	*21*	*4.43 (1.36)*	*4.00*
…is especially helpful when starting or changing a treatment.	*284*	*5.13 (1.17)*	*6.00*	*190*	*5.48 (.88)*	*6.00*	*73*	*4.67 (1.15)*	*5.00*	*21*	*3.57 (1.66)*	*3.00*

…allows patients to ensure that the physician has understood them correctly.	*286*	*5.08 (1.07)*	*5.00*	*192*	*5.28 (.93)*	*6.00*	*73*	*4.97 (.91)*	*5.00*	*21*	*3.67 (1.56)*	*4.00*
…allows a better adherence to medical instructions.	*280*	*5.00 (1.07)*	*5.00*	*189*	*5.29 (.90)*	*6.00*	*70*	*4.67 (.96)*	*5.00*	*21*	*3.52 (1.30)*	*4.00*
…is especially helpful for people with language barriers.	*270*	*4.97 (1.15)*	*5.00*	*182*	*5.22 (1.03)*	*6.00*	*67*	*4.69 (.96)*	*5.00*	*21*	*3.71 (1.59)*	*4.00*
…allows patients to compare their treatment options and make the best decision.	*286*	*4.97 (1.14)*	*5.00*	*193*	*5.33 (.89)*	*6.00*	*73*	*4.45 (.93)*	*5.00*	*20*	*3.30 (1.75)*	*3.00*
…should also be conducted when the diagnosis is communicated during the consultation.	*285*	*4.91 (1.47)*	*6.00*	*192*	*5.28 (1.25)*	*6.00*	*72*	*4.49 (1.36)*	*5.00*	*21*	*3.00 (1.82)*	*3.00*
…is especially helpful for people with cognitive deficits.	*269*	*4.84 (1.31)*	*5.00*	*181*	*5.10 (1.15)*	*6.00*	*68*	*4.46 (1.30)*	*5.00*	*20*	*3.75 (1.77)*	*4.00*
…is especially helpful for older people.	*279*	*4.81 (1.18)*	*5.00*	*188*	*5.01 (1.13)*	*5.00*	*71*	*4.58 (.98)*	*5.00*	*20*	*3.80 (1.64)*	*4.00*
…allows relatives to provide better support to the patient.	*281*	*4.81 (1.15)*	*5.00*	*187*	*5.02 (1.07)*	*5.00*	*73*	*4.58 (1.04)*	*5.00*	*21*	*3.71 (1.42)*	*4.00*
…allows patients to share information with other healthcare professionals.	*284*	*4.76 (1.24)*	*5.00*	*192*	*5.04 (1.11)*	*5.00*	*71*	*4.51 (1.09)*	*5.00*	*21*	*3.05 (1.36)*	*3.00*
…encourages patients to engage with their diagnosis.	*282*	*4.71 (1.17)*	*5.00*	*188*	*5.06 (.93)*	*5.00*	*73*	*4.26 (.99)*	*4.00*	*21*	*3.10 (1.70)*	*3.00*
…is helpful for treatment planning.	*277*	*4.71 (1.29)*	*5.00*	*187*	*5.05 (1.11)*	*5.00*	*69*	*4.22 (1.22)*	*4.00*	*21*	*3.24 (1.45)*	*3.00*
…provides protection for patients and physicians.	*273*	*4.58 (1.42)*	*5.00*	*183*	*4.81 (1.35)*	*5.00*	*70*	*4.33 (1.32)*	*4.00*	*20*	*3.30 (1.60)*	*3.00*
…improves the quality of communication.	*278*	*4.55 (1.25)*	*5.00*	*189*	*4.84 (1.11)*	*5.00*	*69*	*4.19 (1.11)*	*4.00*	*20*	*3.05 (1.57)*	*3.00*
…facilitates patients' active and self-responsible managing of their disease.	*285*	*4.54 (1.28)*	*5.00*	*191*	*4.87 (1.14)*	*5.00*	*73*	*4.12 (1.09)*	*4.00*	*21*	*2.95 (1.60)*	*3.00*
…provides evidence in case of malpractice.	*275*	*4.48 (1.43)*	*5.00*	*185*	*4.65 (1.41)*	*5.00*	*69*	*4.20 (1.40)*	*4.00*	*21*	*3.90 (1.48)*	*4.00*
…facilitates an equal collaboration between patient and physician.	*275*	*4.41 (1.20)*	*4.00*	*186*	*4.69 (1.09)*	*5.00*	*69*	*4.13 (.99)*	*4.00*	*21*	*2.85 (1.46)*	*3.00*

…allows physicians to be more responsive of concerns and needs of patients.	*267*	*4.38 (1.12)*	*4.00*	*176*	*4.57 (1.05)*	*4.00*	*70*	*4.17 (1.06)*	*4.00*	*21*	*3.43 (1.29)*	*3.00*
…leads to physicians taking their patients more seriously.	*271*	*4.31 (1.22)*	*4.00*	*182*	*4.52 (1.16)*	*5.00*	*68*	*4.04 (1.10)*	*4.00*	*21*	*3.33 (1.53)*	*3.00*
…should be made even in brief consultations with little amount of new information.	*287*	*4.17 (1.48)*	*4.00*	*193*	*4.51 (1.38)*	*5.00*	*73*	*3.79 (1.28)*	*4.00*	*21*	*2.38 (1.40)*	*2.00*
…improves the trust between patients and physicians.	*263*	*3.87 (1.13)*	*4.00*	*177*	*4.17 (1.27)*	*4.00*	*65*	*3.43 (1.06)*	*3.00*	*21*	*2.71 (1.38)*	*3.00*
**Levels of agreement towards different statements about concerns regarding consultation recordings**<br>I am concerned…[2,3]												
…that a consultation recording would put pressure on physicians.	*281*	*3.77 (1.34)*	*4.00*	*190*	*3.57 (1.35)*	*4.00*	*71*	*4.03 (1.13)*	*4.00*	*20*	*4.70 (1.49)*	*5.00*
…that physicians would be reserved and less open if consultations were recorded.	*283*	*3.72 (1.45)*	*4.00*	*190*	*3.57 (1.42)*	*4.00*	*72*	*3.85 (1.40)*	*4.00*	*21*	*4.57 (1.57)*	*5.00*
…that the physician-patient-relationship would be more formal if consultations were recorded.	*278*	*3.47 (1.35)*	*4.00*	*188*	*3.24 (1.32)*	*3.00*	*70*	*3.71 (1.23)*	*4.00*	*20*	*4.70 (1.30)*	*5.00*
…that physicians would refer to the recording of the last consultation if any questions came up afterwards.	*284*	*3.45 (1.28)*	*3.00*	*190*	*3.27 (1.22)*	*3.00*	*73*	*3.64 (1.28)*	*4.00*	*21*	*4.33 (1.43)*	*5.00*
…that a consultation recording would be used as evidence against physicians.	*264*	*3.44 (1.48)*	*3.00*	*176*	*3.18 (1.46)*	*3.00*	*68*	*3.76 (1.28)*	*4.00*	*20*	*4.60 (1.54)*	*5.00*
…that relatives could pressure patients into allowing them to listen to their consultation recording.	*278*	*3.41 (1.43)*	*3.00*	*188*	*3.22 (1.36)*	*3.00*	*69*	*3.64 (1.45)*	*4.00*	*21*	*4.38 (1.50)*	*5.00*
…about confidentiality and data protection if consultations were recorded.	*283*	*3.27 (1.57)*	*3.00*	*190*	*2.96 (1.50)*	*3.00*	*72*	*3.72 (1.38)*	*4.00*	*21*	*4.52 (1.83)*	*5.00*
…that listening to the consultation recording would be a psychological burden for patients.	*279*	*3.12 (1.32)*	*3.00*	*190*	*2.85 (1.20)*	*3.00*	*68*	*3.44 (1.26)*	*3.00*	*21*	*4.52 (1.36)*	*5.00*
…about patients perceiving a recording device as stressful during consultations.	*282*	*3.00 (1.35)*	*3.00*	*190*	*2.57 (1.11)*	*3.00*	*71*	*3.55 (1.34)*	*4.00*	*21*	*4.95 (1.02)*	*5.00*

…that patients would be reserved and less open if consultations were recorded.	*285*	*2.99 (1.41)*	*3.00*	*191*	*2.68 (1.24)*	*3.00*	*73*	*3.36 (1.43)*	*3.00*	*21*	*4.57 (1.43)*	*5.00*
…that the technical requirements for making consultation recordings do not exist.	*277*	*2,75 (1.55)*	*3.00*	*188*	*2.61 (1.52)*	*2.00*	*69*	*3.13 (1.49)*	*3.00*	*20*	*2.85 (1.81)*	*3.00*
…that the trust between patients and physicians would decrease if consultations were recorded.	*262*	*2.65 (1.24)*	*3.00*	*177*	*2.41 (1.15)*	*2.00*	*65*	*2.94 (1.17)*	*3.00*	*20*	*3.85 (1.42)*	*4.00*
…that a consultation recording puts too much responsibility on patients.	*281*	*2.60 (1.27)*	*2.00*	*188*	*2.41 (1.17)*	*2.00*	*72*	*2.78 (1.27)*	*3.00*	*21*	*3.71 (1.45)*	*3.00*
…that recording consultations is too complicated for physicians.	*282*	*2.59 (1.44)*	*2.00*	*191*	*2.45 (1.37)*	*2.00*	*71*	*2.83 (1.42)*	*3.00*	*20*	*3.05 (1.99)*	*3.00*
…that recording consultations is too complicated for patients.	*283*	*2.53 (1.36)*	*2.00*	*192*	*2.28 (1.22)*	*2.00*	*71*	*3.04 (1.38)*	*3.00*	*21*	*3.05 (1.91)*	*3.00*
…that the quality of the communication decreases through a consultation recording.	*276*	*2.39 (1.32)*	*2.00*	*187*	*2.05 (1.19)*	*2.00*	*68*	*2.90 (1.22)*	*3.00*	*21*	*3.71 (1.45)*	*4.00*

Notes:

[1]answers were assessed on a 6-point Likert scale, ranging from *very negative* (=1) to *very positive* (=6);

[2]answers were assessed on a 6-point Likert scale, ranging from *completely disagree* (=1) to *completely agree (=6*);

[3]Items are ordered from highest to lowest agreement
